# Epigenetic switch drives the conversion of fibroblasts into proinvasive cancer-associated fibroblasts

**DOI:** 10.1038/ncomms10204

**Published:** 2015-12-15

**Authors:** Jean Albrengues, Thomas Bertero, Eloise Grasset, Stephanie Bonan, Majdi Maiel, Isabelle Bourget, Claude Philippe, Cecilia Herraiz Serrano, Samia Benamar, Olivier Croce, Victoria Sanz-Moreno, Guerrino Meneguzzi, Chloe C. Feral, Gael Cristofari, Cedric Gaggioli

**Affiliations:** 1 INSERM U1081, CNRS UMR7284, Institute for Research on Cancer and Aging, Nice (IRCAN), University of Nice Sophia Antipolis, Medical School, 28 Avenue Valombrose, Nice F-06107, France; 2 Tumour Plasticity Laboratory, Randall Division of Cell and Molecular Biophysics, New Hunt's House, Guy's Campus, King's College London, London SE1UL, UK; 3 CNRS UMR 7278, IFR48, Unité de Recherche sur les Maladies Infectieuses et Tropicales Emergentes, Faculté de Médecine, Aix-Marseille Université, 13385 Marseille Cedex 05, France

## Abstract

Carcinoma-associated fibroblasts (CAF) mediate the onset of a proinvasive tumour microenvironment. The proinflammatory cytokine LIF reprograms fibroblasts into a proinvasive phenotype, which promotes extracellular matrix remodelling and collective invasion of cancer cells. Here we unveil that exposure to LIF initiates an epigenetic switch leading to the constitutive activation of JAK1/STAT3 signalling, which results in sustained proinvasive activity of CAF. Mechanistically, p300-histone acetyltransferase acetylates STAT3, which, in turn, upregulates and activates the DNMT3b DNA methyltransferase. DNMT3b methylates CpG sites of the SHP-1 phosphatase promoter, which abrogates SHP-1 expression, and results in constitutive phosphorylation of JAK1. Sustained JAK1/STAT3 signalling is maintained by DNA methyltransferase DNMT1. Consistently, in human lung and head and neck carcinomas, STAT3 acetylation and phosphorylation are inversely correlated with SHP-1 expression. Combined inhibition of DNMT activities and JAK signalling, *in vitro* and *in vivo*, results in long-term reversion of CAF-associated proinvasive activity and restoration of the wild-type fibroblast phenotype.

## Abstract (web summary)

Carcinoma-associated fibroblasts are key components of solid tumours and associated with poor clinical outcome. Here the authors show that the cytokine LIF initiates an epigenetic switch which results in the sustained invasive activity of the tumour cells.

## Graphical abstract

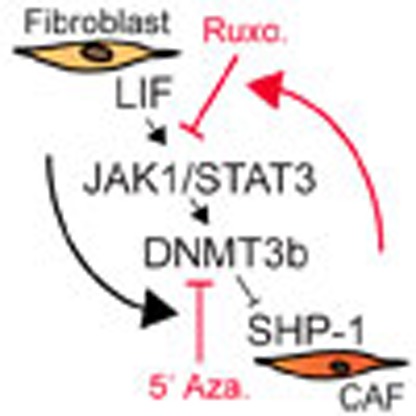

Carcinoma-associated fibroblasts (CAF) are key components of solid tumour ecosystems, including breast, skin, head and neck, prostate and lung cancers^1^,^2^,^3^. CAF play an active role in tumour development and their presence within the tumour stroma is associated with poor clinical outcome^4^,^5^,^6^,^7^,^8^. Besides promoting primary tumours, CAF remodel the tumour microenvironment by generation of tensile forces within the extracellular matrix (ECM) that support favourable cues for tumour invasion^9^,^10^,^11^,^12^. CAF-dependent excessive ECM remodelling leads to tumour fibrosis, which is associated with tumour cell dissemination and metastasis^13^,^14^. CAF-dependent profibrotic activity is governed by constitutive activation of the Janus kinase 1 (JAK1), which leads to constitutive phosphorylation of its downstream target, the STAT3 (signal transducer and activator of transcription 3) transcription factor^15^. We have demonstrated that the proinflammatory LIF (leukaemia inhibitory factor) cytokine initiates the sustained proinvasive stromal fibroblast phenotype observed in cancers^16^. However, how the activated status of stromal fibroblast is sustained remains to be elucidated.

STAT3 belongs to a large family of transcription factors, and its constitutive activation has been observed in 70% of solid and haematological tumours^17^. STAT3-dependent transcriptional activity is mediated by phosphorylation on tyrosines 705 and 727 by the JAK family kinases in response to cytokine-mediated signalling^18^. Activated STAT3 translocates to the nucleus and binds promoter DNA sequences to regulate targeted gene transcription. STAT3 activity is enhanced by acetylation on lysine 685 by the histone acetyltransferases (HAT) p300/CREB-binding protein (CBP), a post-transcriptional modification that is reversed by histone deacetylases (HDACs)^19^,^20^,^21^. Acetylated STAT3 binds to the DNA methyltransferase DNMT1, which mediates targeted gene silencing by methylation of promoter DNA sequences^22^. DNA methylation plays critical roles in control of sustained and constitutive activation of signalling pathways^23^. Overall, both tumour and stromal cell DNA are hypomethylated; however, hypermetylation of CpG dinucleotides in gene promoters is known to silence tumour-suppressor gene expression^24^,^25^,^26^,^27^,^28^. Among the major DNA methyltransferases (DNMTs), DNMT1 maintains pre-existing DNA methylation patterns along with DNA replication and cell division; DNMT3a and DNMT3b target unmethylated CpGs and introduce *de novo* methylations^29^.

Here using combination of three-dimensional model of organotypic invasion assays of head and neck and breast tumours, and using *in vitro* and *in vivo* models of breast carcinomas, we demonstrate that an epigenetic switch initiates and maintains the proinvasive phenotype of CAF. We show that LIF induces constitutive activation of the JAK1/STAT3 signalling pathway by post-translational regulation of STAT3 acetylation by p300, in fibroblasts. Acetylated STAT3 leads to an epigenetic-dependent loss of expression of the SHP-1 tyrosine phosphatase, which is a negative regulator of the JAK/STAT pathway. Silencing of SHP-1 by promoter methylation leads to sustained constitutive phosphorylation of the JAK1 kinase and the STAT3 transcription factor that maintain the contractile and proinvasive fibroblasts abilities. Blockage of both JAK signalling and DNA methyltransferase activities both *in vitro* and *in vivo* results in long-term proinvasive phenotypic reversion of CAF. Finally, we corroborate our *in vitro* and *in vivo* findings through immunhistological analysis of STAT3 activity and SHP-1 expression in both head and neck and lung human carcinoma biopsies. Therefore, we conclude that in human carcinomas from different origins, LIF induces a sustained proinvasive activation of CAF through an epigenetic-dependent loss of SHP-1 phosphatase.

## Results

### Epigenetic mechanisms sustain the proinvasive CAF phenotype

**Figure 1:**
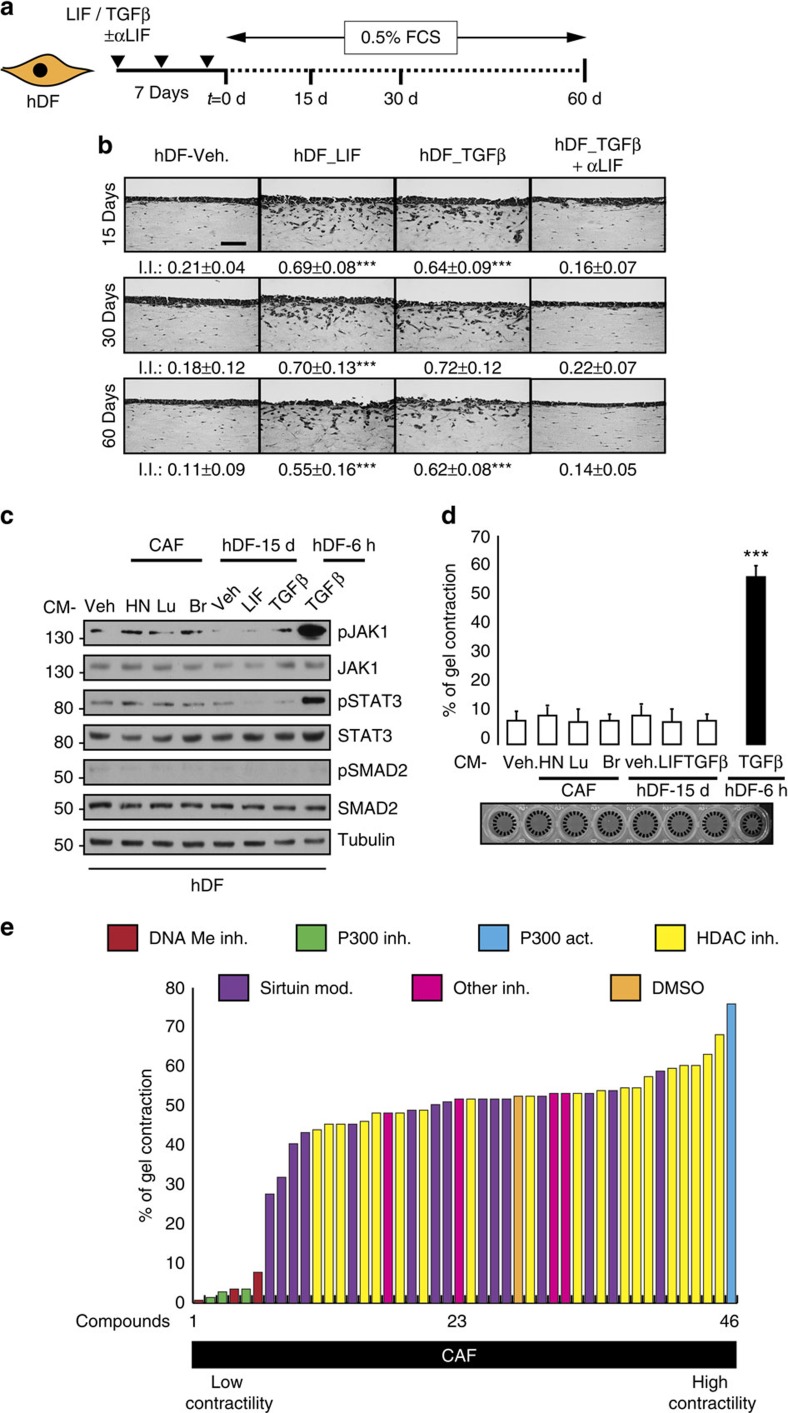
Epigenetic-dependent proinvasive fibroblasts phenotype. (**a**) Schematic representation of experimental conditions for long-term maintenance of the proinvasive properties of LIF- and TGFβ1-activated fibroblasts. (**b**) Representative images of haematoxylin and eosin (H&E) coloration of paraffin-embedded sections of SCC12 in response to control hDF (Veh.) or hDF previously activated for 7 days with LIF or TGFβ1, in presence or absence of LIF blocking antibody (αLIF) after 15, 30 and 60 days of culture in 0.5% SVF media. Scale bar, 100 μm. I.I., invasion index (*n*=3; mean±s.d.; ***P<0.001). (**c**) Immunoblot of pJAK1, pSTAT3 and pSMAD2 in hDF cells stimulated by three CAF-CM, long-term LIF and TGFβ1-activated hDF-CM and short-term TGFβ-activated hDF-CM. Immunoblot of JAK1, STAT3, SMAD2 and tubulin as control. (**d**) Percentage of gel contraction by hDF stimulated by three CAF-CM, long-term LIF and TGFβ1-activated hDF-CM and short-term TGFβ-activated hDF-CM (*n*=3 in triplicate, mean+s.d., ***P<0.001). (**e**) Quantification of matrix remodelling by CAF in the presence of small molecules inhibitors for 6 days. (*n*=3 in triplicates).

We first assessed whether, similar to CAF isolated from head and neck, lung and breast human carcinomas (HN-CAF, Lu-CAF and Br-CAF, respectively), the long-term LIF or TGFβ-activated human dermal fibroblasts (hDF_LIF or hDF_TGFβ) constitutively retain their proinvasive properties. hDF were stimulated for 7 days in culture using LIF or TGFβ in the presence or absence of a LIF blocking antibody. After 15- 30- and 60-day culture in low serum concentration (Fig. 1a), the hDF proinvasive ability was assessed using a three-dimensional SCC12 cell organotypic invasion assay^30^. In these conditions LIF induced the sustained fibroblast proinvasive phenotype (Fig. 1b) and, constitutively, the JAK1/STAT3 signalling pathway (Supplementary Fig. 1a) as observed with CAF compared with primary hDF (Supplementary Fig. 1b). Interestingly, TGFβ, the major *in vitro* and *in vivo* CAF activator, relied on LIF to constitutively activate the proinvasive ability of hDF (Fig. 1b) and the JAK1/STAT3 signalling pathway (Supplementary Fig. 1a). These results indicate that a 7-day LIF stimulation is sufficient to confer a long-term proinvasive CAF-like phenotype to hDF. CAF secrete procarcinogenic factors, including interleukin-6 (IL6)-family cytokines^2^ that may sustain constitutive activation of JAK1 kinase via an autocrine regulatory loop. Therefore, we investigated whether media conditioned (CM) by CAF, hDF_LIF and hDF_TGFβ can activate JAK1/STAT3 signalling in hDF. Stimulation of hDF by long-term CAF, LIF- and TGFβ-activated hDF CM failed to promote JAK1/STAT3 phosphorylation (Fig. 1c) and collagen gel contraction (Fig. 1d), while short-term TGFβ-activated CM (hDF-TGFβ-6 h), in which LIF is detectable^16^, promoted both STAT3 phosphorylation and gel contraction (Fig. 1c,d). Accordingly, in hDF_LIF, abrogation of GP130 receptor or addition of a specific LIF blocking antibody failed to alter STAT3 phosphorylation, which contrasts with the abrogation of JAK1 expression (Supplementary Fig. 1c,d). Taken together, these data demonstrate that LIF confers permanent proinvasive conversion to hDF that is independent of a LIF autocrine signalling regulatory loops but dependent on JAK1 activity. We thus hypothesized that epigenetic modifications might be involved in the constitutive activation of CAF and hDF_LIF. To test this idea, HN-CAF-dependent three-dimensional matrix gel contraction assays were performed in the presence of 45 small-molecule inhibitors targeting the epigenetic and DNA-modifier cell machinery (Fig. 1e, Supplementary Fig. 1e and Supplementary Table 1). For each tested molecule, the screen was performed at optimized concentrations (Supplementary Table 1); dimethylsulphoxide was used as a control. Among the six classes of inhibitors, three HAT p300 inhibitors (Garcinol, Anacardic acid and C646) and three DNA methyltransferase inhibitors (5′-Aza-2′-deoxycytidine, Zebularine and Decitabine) blocked the CAF contractile capacity (Fig. 1e). Accordingly, the p300 activator CTPB (*N*-(4-chloro-3-trifluoromethyl-phenyl)-2-ethoxy-benzamide) significantly increased CAF contractility (Fig. 1e). Inhibition of matrix remodelling by both p300 and DNMT inhibitors was further confirmed in hDF_LIF (Supplementary Fig. 1e). In light of these results, the role of p300 in CAF- and hDF-LIF-dependent SCC12 cell invasion was further investigated. Using C646, a small-molecule inhibitor, the crucial role of p300 in proinvasive activity was established (Supplementary Fig. 1f,g). These results demonstrate that LIF governs the long-term activation of proinvasive fibroblasts, which also involves DNMTs and p300 activities, and further suggests that the sustained phenotypes observed in CAF rely on epigenetic modifications.

### P300 mediates STAT3 acetylation for fibroblast activation

**Figure 2:**
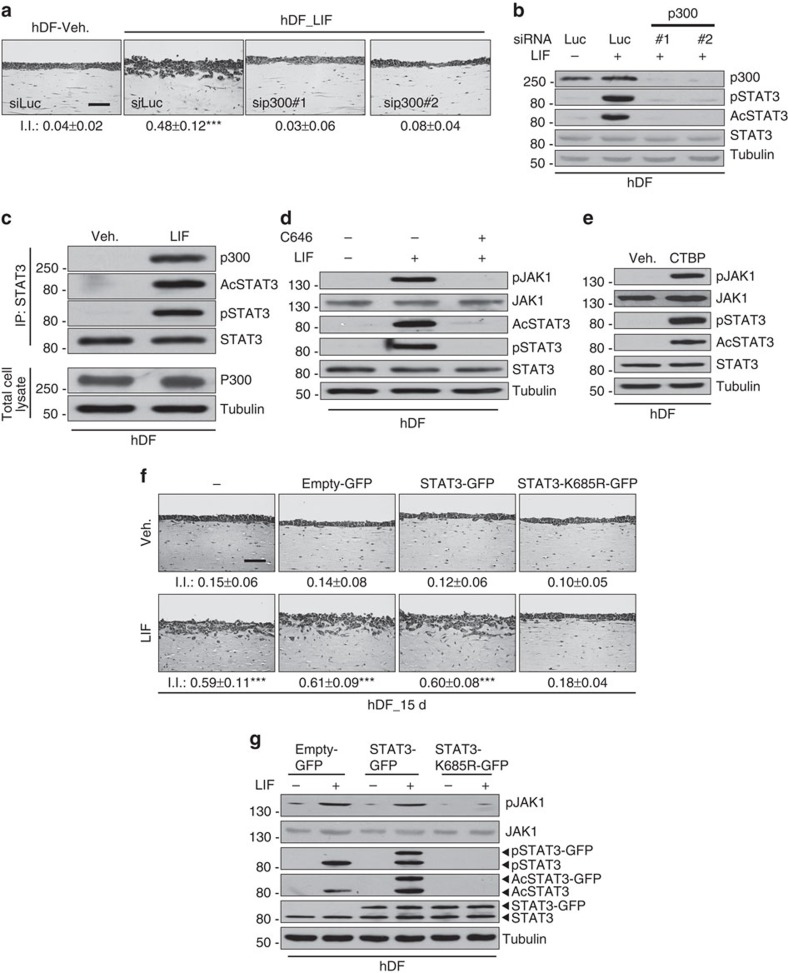
P300 acetylates STAT3 for proinvasive CAF activity. (**a**) Representative images of H&E coloration of paraffin-embedded sections of SCC12 in response to control hDF transfected with control siRNA (siLuc) or p300 (sip300) prior to control (veh.) or LIF stimulation. Scale bar, 100 μm (*n*=3; mean ±s.d.; ***P<0.001). (**b**) Immunoblot of p300, AcSTAT3 and pSTAT3 in hDF transfected with RNAi control (siLuc) or targeting p300 (siP300#1 and #2) before LIF stimulation. Immunoblot of STAT3 and tubulin as control. (**c**) Immunoblot of p300, AcSTAT3, pSTAT3 and STAT3 in hDF following immunoprecitation of STAT3 on 24 h LIF stimulation. Immunblot of p300 and tubulin in total lysate as control. (**d**) Immunoblot of pJAK1, AcSTAT3 and pSTAT3 in hDF in presence or absence of C646 inhibitor followed by long-term control (veh.) or LIF stimulation. Immunoblot of JAK1, STAT3 and tubulin as control. (**e**) Immunoblot of pJAK1, AcSTAT3 and pSTAT3 in control or long-term CTBP stimulated hDF. Immunoblot of JAK1, STAT3 and tubulin as control. (**f**) Representative images of H&E colouration of paraffin-embedded sections of SCC12 in response to control hDF (veh.) and hDF_LIF overexpressing a control (empty-GFP) or a wild-type STAT3 (STAT3-GFP) or acetylated-deficient STAT3 mutant (STAT3-K685R-GFP). Scale bar, 100 μm (I.I., invasion index; *n*=3; mean ±s.d.; ***P<0.001). (**g**) Immunoblot of pJAK1, pSTAT3 and AcSTAT3 in hDF overexpressing a control vector (empty-GFP), a wild-type (WT) STAT3 (STAT3-GFP) or acetylated-deficient STAT3 mutant (STAT3-K685R-GFP) in response to long-term LIF stimulation. Immunoblot of JAK1, STAT3, and tubulin as control.

Screening for small-molecule inhibitors of the epigenetic cell machinery identified p300 as a crucial regulator of proinvasive fibroblasts matrix remodelling *in vitro*. Indeed, in hDF, RNA interference (RNAi)-mediated silencing of p300 before LIF initiation resulted in blockage of the sustained proinvasive property displayed by hDF_LIF (Fig. 2a,b). Interestingly, silencing of p300 in HN-CAF resulted in loss of their proinvasive potential (Supplementary Fig. 2a,b), which confirmed the results obtained in the contractility screens (Fig. 1e, Supplementary Fig. 1c). Furthermore, the p300 small-molecule activator CTBP was sufficient to promote hDF-dependent matrix remodelling (Supplementary Fig. 2c) and the sustained proinvasive cell capacity (Supplementary Fig. 2d). Taken together, these data showed that p300 plays a central role in the proinvasive fibroblast activation. Besides acetylating histones, p300 also acetylates STAT3, which is crucial for STAT3 transcriptional activity^21^. We then showed that in hDF, LIF induced p300 binding to activate STAT3 (Fig. 2c) and RNAi silencing of p300 or blockage of p300 activity by the C646 small-molecule inhibitor inhibited the LIF-dependent constitutive activation of JAK1 phosphorylation and both the STAT3 phosphorylation on residue tyrosine 705 and the acetylation on residue lysine K685 (Fig. 2b,d). Furthermore, activation of p300 by CTPB was sufficient to constitutively activate the JAK1/STAT3 signalling pathway in hDF cells (Fig. 2e). This observation further confirms that p300 is responsible for STAT3 acetylation on LIF-dependent proinvasive fibroblast activation and suggests that STAT3 acetylation is pivotal in this process. At this stage, the role of STAT3 acetylation in sustained fibroblast proinvasive activity was then assessed. Using organotypic invasion assays, hDF stably expressing either green fluorescent protein (GFP; empty-GFP), or the wild-type form of STAT3 (STAT3-GFP) or a non-acetylated STAT3 mutant fused to GFP (STAT3-K685R-GFP), were investigated for their LIF-dependent sustained proinvasive capacity. HDF_LIF overexpressing either the control or the WT-STAT3 vector were found to retain proinvasive properties, that were abrogated in cells expressing a non-acetylated form of STAT3 (Fig. 2f). Furthermore, molecular analysis of the JAK1/STAT3 signalling pathway in hDF_LIF expressing the mutant STAT3-K685R cDNA, revealed that STAT3 acetylation is compulsory for phosphorylation of the residue tyrosine 705, and that LIF-dependent JAK1/STAT3 constitutive phosphorylation requires STAT3 acetylation (Fig. 2g). HN-CAF expressing the mutant STAT3-K685R cDNA, lost proinvasive activity (Supplementary Fig. 2e) and STAT3 phosphorylation, independent of JAK1 activity (Supplementary Fig. 2f). Taken together, these data demonstrate that STAT3 acetylation on residue lysine 685 is compulsory for proinvasive fibroblast property.

### DNMTs govern LIF-dependent fibroblasts activation

**Figure 3:**
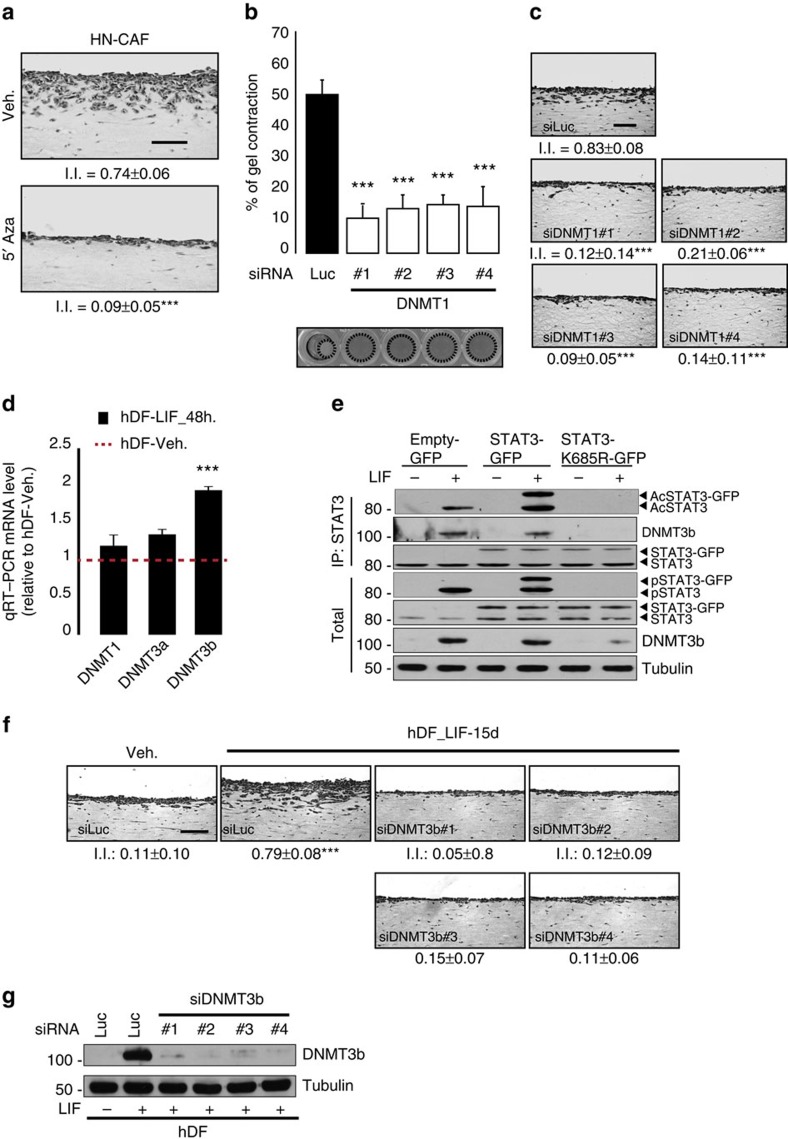
DNMTs controls proinvasive fibroblasts' activity. (**a**) Representative images of H&E colouration of paraffin-embedded sections of SCC12 in response to CAF in the absence (Veh.) or presence of 5′-Aza inhibitor (*n*=3, I.I., invasion index, mean±s.d., ***P<0.001). Scale bar 100 μm. (**b**) Percentage of gel contraction by CAF transfected with siRNA control (siLuc) or targeting DNMT1 (siDNMT1#1, #2, #3, #4) (*n*=3 in triplicate, mean+s.d., ***P<0.001). Bottom images show the contracted gels. (**c**) Representative images of H&E colouration of paraffin-embedded sections of SCC12 in response to CAF transfected with siRNA control (siLuc) or targeting DNMT1 (siDNMT#1, #2, #3, #4) (*n*=3, mean±s.d., ***P<0.001). Scale bar 100 μm. (**d**) Quantification of mRNA level of DNMT1, DNMT3a and DNMT3b in hDF following 48 h stimulation of LIF relative to control hDF (red dotted line) (*n*=3 in triplicate, mean+s.d., ***P<0.001). (**e**) Immunoblot of AcSTAT3, DNMT3b and STAT3 in hDF cells overexpressing a control or a wild type STAT3 or acetylated deficient STAT3 mutant following STAT3 immunoprecipitation upon LIF stimulation. Immunoblot of pSTAT3, STAT3 and tubulin on total lysates as control. (**f**) Representative images of H&E coloration of paraffin-embedded sections of SCC12 in response to hDF transfected with siRNA control (siLuc) or targeting DNMT3b (siDNMT3b#1 and #2) and subsequently long-term control- (Veh.) or LIF-activated hDF (*n*=3, mean±s.d., ***P<0.001). Scale bar 100 μm. (**g**) Immunoblot of DNMT3b in hDF activated or not by LIF transfected with siRNA control (siLuc) or targeting DNMT3b (siDNMT3b#1, #2, #3, #4). Immunoblot of tubulin as control.

Screening using the small-molecule inhibitor library also identified hypomethylating agents (DNA methylation inhibitors) as the most potent molecules blocking HN-CAF and hDF_LIF matrix remodelling (Fig. 1e, Supplementary Fig. 1c). We disclosed that 5′-azacytidine (5′-Aza), decitabine and zebularine, pyrimidine nucleoside analogues of cytidine incorporated into DNA, where it reversibly inhibits DNMT, interfered with CAF-dependent SCC12 cell collective invasion (Fig. 3a). Therefore, we next assessed the role of the DNMT protein family in LIF-dependent constitutive fibroblast activation in three-dimensional *in vitro* organotypic invasion assays. Specific RNAi-dependent silencing of DNMT1 expression in fibroblasts (Supplementary Fig. 3a) demonstrated that DNMT1 expression is required for CAF-dependent matrix remodelling (Fig. 3b) and invasion of SCC12 cells (Fig. 3c). DNMT1 is primarily implicated in the maintenance of CpG methylation throughout development and cell divisions, while DNMT3 role consists in *de novo* CpG methylation^31^. Therefore, to assess a possible involvement of DNMT3 in initiation of the contractile and proinvasive process of the LIF-dependent fibroblast activation, we investigated the messenger RNA (mRNA) steady-state level of the DNMT family after short-term LIF stimulation of hDF. We first found that mRNA steady-state level of DNMT3b, but not that of DNMT1 or 3a, was significantly induced after short-term LIF stimulation of hDF (Fig. 3d). We second observed that DNMT3b binds to STAT3 active form upon LIF stimulation (Fig. 3e); therefore, the potential role for DNMT3b in induction of the constitutive proinvasive activation by LIF in hDF was investigated. Specific deletion of DNMT3b expression before LIF stimulation resulted in blockage of LIF-dependent activation of a proinvasive phenotype in hDF (Fig. 3f,g); whereas loss of DNMT3b expression in CAF and in long-term activated hDF_LIF had no effect on gel contraction (Supplementary Fig. 3b,c). Taken together, these results demonstrate that DNMT3b governs the LIF-dependent proinvasive fibroblast activation, which in HN-CAF is sustained by DNMT1. This explains why in HN-CAF, which have undergone the activation step, mediated by a LIF/DNMT3b signalling, targeting DNMT3b is ineffective, while targeting DNMT1 blocks the proinvasive phenotype.

### JAK1/STAT3 sustained activity is mediated by loss of SHP-1

**Figure 4:**
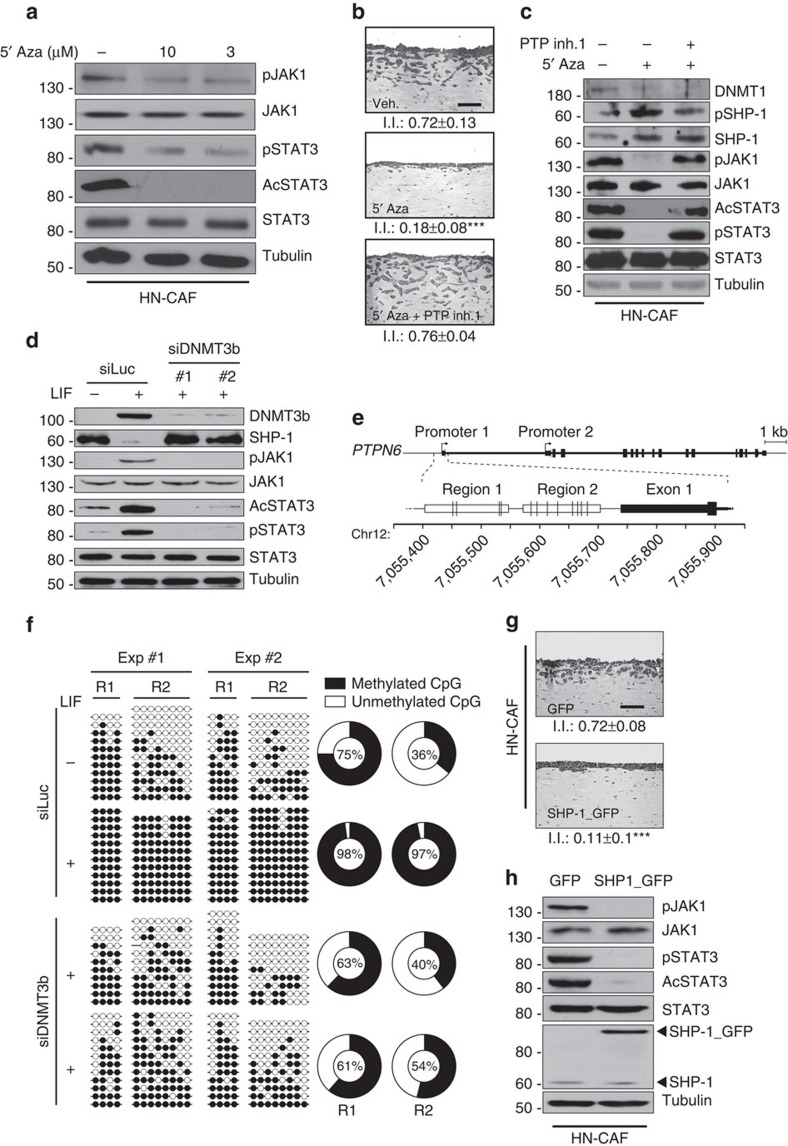
Epigenetic-dependent sustained JAK1/STAT3 signalling in CAF. (**a**) Immunoblot of pJAK1, pSTAT3 and AcSTAT3 in CAF after 7-days treatment in the presence or absence of 5′-Aza inhibitor at 10 and 3 μM final concentration. Immunoblot of JAK1, STAT3 and tubulin as control. (**b**) Representative images of H&E coloration of paraffin-embedded sections of SCC12 in response to CAF following 5′-Aza inhibitor alone or in combination with PTP inhibitor 1 (*n*=3, mean±s.d., ***P<0.001). (**c**) Immunoblot of DNMT1, pSHP-1, SHP-1, pJAK1, pSTAT3 and AcSTAT3 in CAF following 5′-Aza inhibitor alone or in combination with PTP inhibitor 1. Immunoblot of JAK1, STAT3 and tubulin as control. (**d**) Immunoblot of DNMT3b, SHP-1, pJAK1, pSTAT3 and AcSTAT3 in hDF activated or not by LIF transfected with siRNA control (siLuc) or siRNA targeting DNMT3b (siDNMT3b#1, #2). Immunoblot of JAK1, STAT3 and tubulin as control. (**e**) Schematic illustration of the *PTPN6* gene structure and the regions analysed by bisulfite sequencing. The *PTPN6* gene has two alternative promoters. We restricted our analysis to Promoter 1 since Promoter 2 is mostly active in the haematopoietic lineage. Vertical lines in Region 1 and Region 2 indicate the position of the CpG dinucleotides. (**f**) Bisulfite-sequencing results in two regions of the *PTPN6* promoter (R1 and R2) in hDF transfected with control RNAi (siLuc) and RNAi targeting DNMT3b (siDNMT3b#1 and #2) and subsequently long-term control (−) or LIF-activated (+). Each line represents an individual sequence. Open and closed circles denote unmethylated and methylated CpG dinucleotides, respectively. Donut charts summarize the global proportion of methylated CpG with the percentage in the center (*n*=2, *P*<0.0001, two-tailed Fisher's exact test). (**g**) Representative images of H&E coloration of paraffin-embedded sections of SCC12 in response to CAF overexpressing control vector (GFP) or SHP-1 gene (SHP-1_GFP) (*n*=3, mean±s.d., ***P<0.001). (**h**) Immunoblot of SHP-1, pJAK1, pSTAT3 and AcSTAT3 in CAF overexpressing control vector (GFP) or SHP-1 gene (SHP-1_GFP). Immunoblot of JAK1, STAT3 and tubulin as control.

Having shown that restriction of the DNMTs catalytic activities inhibits both the establishment and the maintenance of the proinvasive phenotype of carcinoma- and LIF-activated fibroblast, we first investigated the molecular mechanisms underlying the DNMT-dependent proinvasive fibroblast activation. The effect of 5′-Aza on the JAK1/STAT3 constitutive signalling pathway activity was thus assessed after treatment of HN-CAF and LIF-activated fibroblast. Inhibition of both STAT3 phosphorylation and acetylation was observed (Fig. 4a) potentially consequent to inhibition of JAK1 phosphorylation on residue tyrosine 1022 (Fig. 4a, Supplementary Fig. 4a), which reflects its kinase activity^32^. JAK1 tyrosine 1022 phosphorylation is controlled by a balance between JAK kinase transactivation and dephosphorylation^33^. To address the functional role of 5′-Aza in the JAK1/STAT3 signalling activity and by consequence in HN-CAF proinvasive ability, we hypothesized that a DNMT-dependent epigenetic silencing regulates JAK1 phosphatase expression. Thus HN-CAF were exposed to 5′-Aza and then to orthovanadate, a broad phosphatase inhibitor. Under such experimental conditions JAK1 phosphorylation and both STAT3 phosphorylation and acetylation were rescued (Supplementary Fig. 4b), which validated the working hypothesis. Since tyrosine-protein phosphatase non-receptor type 6 (PTPN6), also known as SHP-1 (Src homology region 2 domain-containing phosphatase-1), is known to dephosphorylate a number of tyrosine kinases including JAK1 (ref. 34), the possibility that SHP-1 could regulate the proinvasive activity of HN-CAF in our system was assessed. Indeed, specific SHP-1 inhibition by PTP inhibitor 1 (PTP inh 1) restored the proinvasive property blocked by the 5′-Aza treatment (Fig. 4b). Subsequent analyses at the molecular level demonstrated that SHP-1 re-expression induced by 5′-Aza treatment correlated with decreased JAK1/STAT3 activity in HN-CAF, and that inhibition of SHP-1 phosphatase activity restored a constitutive JAK1/STAT3 signalling activity (Fig. 4c). Furthermore, in both HN-CAF and hDF_LIF, inhibition of both the proinvasive capacity (Supplementary Fig. 4c) and JAK1/STAT3 signalling (Supplementary Fig. 4d,e) induced by loss of DNMT1 expression consequent to RNAi treatment was rescued by specific RNAi targeting of SHP-1 expression. Taken together, these data demonstrate that long-term activation of proinvasive fibroblast activity is sustained by constitutive activation of the JAK1/STAT3 signalling pathway through a DNMT1-dependent downregulation of the SHP-1 phosphatase expression. At the initiation phase, LIF induces an enhanced expression of DNMT3b and the loss of SHP-1 expression, which results in constitutive JAK1/STAT3 signalling pathway activity. Accordingly blockage of DNMT3b expression, prior to LIF stimulation, leads to inhibition of JAK1/STAT3 phosphorylation and rescue of SHP-1 protein expression (Fig. 4d). Consistently with our previous data, inhibition of p300 catalytic activity or loss of p300 expression consequent to RNAi treatment before LIF stimulation inhibits the loss of SHP-1 expression in hDF (Supplementary Fig. 4f,g). A possible explanation is that LIF, through a DNMT-dependent pathway, regulates the methylation status of the *SHP-1* promoter. To address this possibility, we analysed by bisulfite sequencing the two regions R1 and R2 of the *PTPN6* promoter 1 (ref. 35) rich in CpG dinucleotides (depicted in Fig. 4e). Strikingly, long-term LIF-treated hDF compared with untreated controls, presented hypermethylation of the *PTPN6* promoter, a DNMT3b- (Fig. 4f, Supplementary Fig. 4h) dependent modification that was hampered by siRNA-mediated knockdown of DNMTs during LIF stimulation. Accordingly, transgenic expression of SHP-1 in HN-CAF was sufficient to block the proinvasive capacity of these cells (Fig. 4g) and to inhibit their JAK1/STAT3 signalling pathway activity (Fig. 4h). Accordingly, SHP-1 was undetectable in CAF from multiple carcinoma origins compared with three primary hDF (Supplementary Fig. 4i) and regions 1 and 2 of *PTPN6* promoter 1 of human CAF were hypermethylated compared with the hDF *PTPN6* promoter (Supplementary Fig. 4j). These results establish that a LIF/p300/DNMT signalling governs the initial step of JAK1/STAT3 constitutive activation through *SHP-1* silencing. Once this epigenetic repression is established, targeting DNMT3b has no effect, while targeting DNMT1 inhibits the proinvasive phenotype of CAF.

### Phenotypic and molecular constitutive reversion of CAF

**Figure 5:**
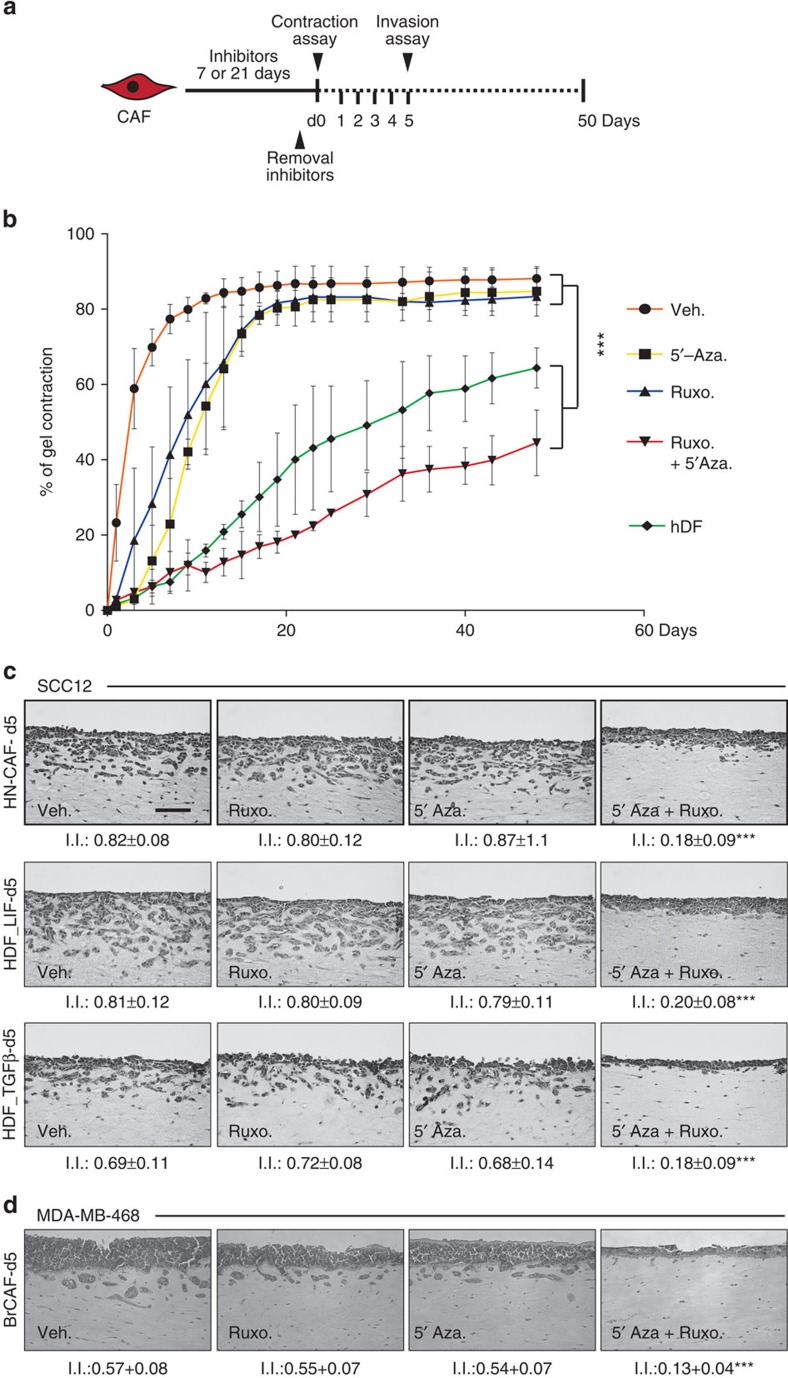
Phenotypic and molecular reversion of CAF. (**a**) Schematic representation of the experimental conditions for long-term phenotypic and molecular reversion of the proinvasive property of activated fibroblasts. After inhibitors treatment for 7 or 21 days, cells are embedded in matrix for a 50-day gel contraction assay, assayed every day for a period of 5 days for molecular analysis, and assayed at day 5 after inhibitors removal for proinvasive ability. (See also Supplementary Fig. 5). (**b**) Percentage of gel contraction of hDF and CAF for a period of 50 days following a 7-day treatment of either Ruxolitinib or 5′-Aza or both inhibitors together. (**c**) Representative images of H&E coloration of paraffin-embedded sections of SCC12 in response to CAF, hDF_LIF and hDF_TGFβ1, 5 days after a period of 7-day treatment of Ruxolitinib or 5′-Aza or both inhibitors together (*n*=3, mean±s.d., ***P<0.001). Scale bar 100 μm. (**d**) Representative images of H&E colouration of paraffin-embedded sections of MDA-MB-468 human breast cancer cell line in response to breast CAF 5 days after a period of 7-day treatment of Ruxolitinib or 5′-Aza or both inhibitors together (*n*=3, mean±s.d., ***P<0.001).

**Figure 6:**
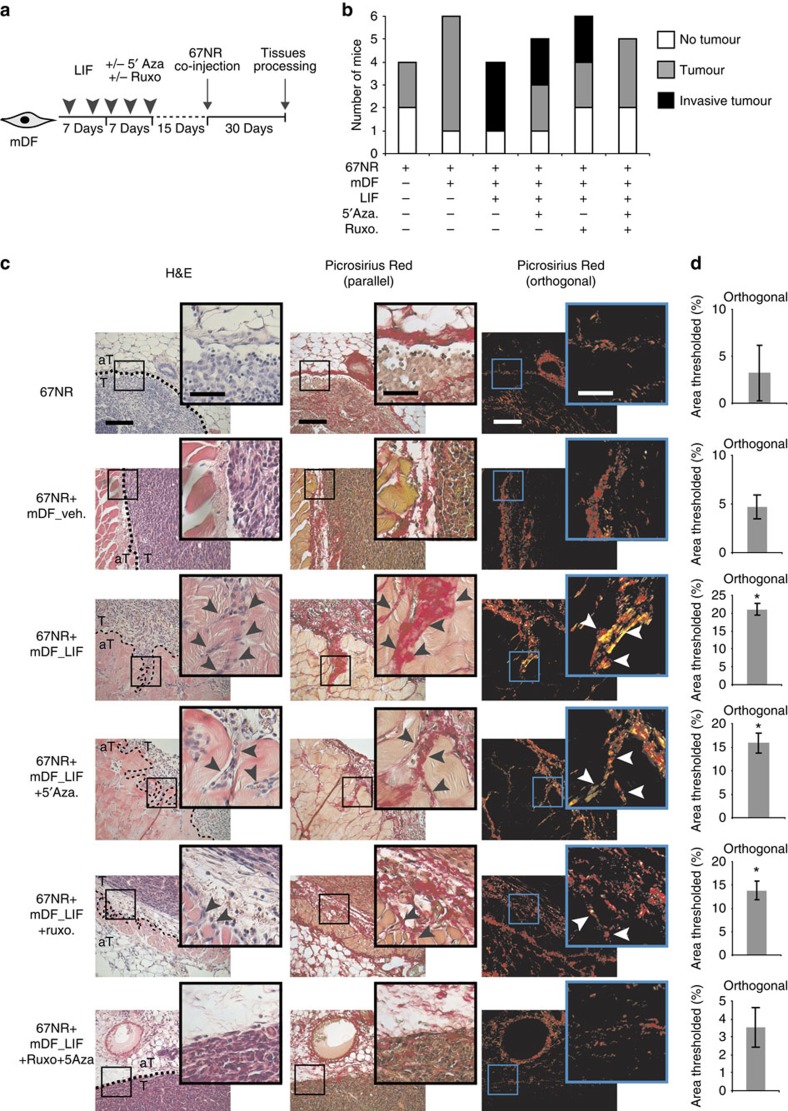
Stromal DNMT and JAK favour tumour invasion *in vivo*. (**a**) Schematic representation of the experimental conditions used for *in vivo* cell co-implantation into the mammary fat pad of 8-week-old BALB/c female mice. 67NR mouse breast carcinoma cells were co-injected together with LIF preactivated mDF and treated with inhibitors. (**b**) Graphic representation of tumour formation induced by 67NR cells alone or in the presence of LIF-preactivated mDF. Total numbers of mice bearing tumours after single or combined inhibitor treatment are shown. (**c**) H&E coloration of paraffin-embedded sections of primary tumours isolated from mice (left panels) showing 67NR cells invading from the primary tumour (T) into the adjacent tissue (aT) (black arrow). Picrosirius Red staining visualized by both parallel (middle panels) and orthogonal (right panels) light showing tumour ECM remodelling at the areas invaded by the tumoral 67NR cells. Scale bars, 200 and 100 μm for × 20 and × 40X magnifications, respectively. (**d**) Quantification of Picrosirius Red staining using ImageJ software. Percentage of threshold area is shown (mean±s.d.; *P<0.05).

Ruxolitinib, an FDA approved JAK1/2 pharmacological inhibitor, targets proinvasive tumour microenvironment modifications by transiently inhibiting human CAF contractility^16^. Having demonstrated here that DNMT1 supports constitutive JAK1/STAT3 activation and proinvasive activity of HN-CAF, we hypothesized that treatment of activated fibroblasts with 5′-Aza could permanently revert the activated phenotype. To test such an idea, activated fibroblasts were cultured for 7 days in the presence of either Ruxolitinib or 5′-Aza. After removal of the inhibitors, the molecular activity of the JAK1/STAT3 signalling pathway was measured for 5 days, and the contractile activity of the treated cells was monitored for additional 45 days in collagen gel (Fig. 5a). HN-CAF treated with Ruxolitinib restored STAT3 phosphorylation 2 days after drug removal, while SHP-1 expression was not affected (Supplementary Fig. 5a). 5′-Aza treatment resulted in a transient 3-day decrease of STAT3 phosphorylation, SHP-1 expression was first induced on DNMT1 inhibition, but restoration of the STAT3 activity correlated with the loss of SHP-1 protein (Supplementary Fig. 5b). Taken together, these data indicate that in HN-CAF the effects of both Ruxolitinib and 5′-Aza are transient. Because both drugs act at different signalling levels to regulate the JAK1/STAT3 signalling pathway, possible synergistic action of the two compounds was explored. Combined 7-day Ruxolitinib and 5′-Aza treatment of activated fibroblasts resulted in long-term abrogation of the JAK1/STAT3 phosphorylation and rescued SHP-1 protein expression (Supplementary Fig. 5c). Further, such long-term abrogation of the JAK1/STAT3 signalling pathway activity was confirmed using hDF_LIF (Supplementary Fig. 5d) by dual inhibitor treatment. We next assessed whether the combined treatment could revert activated fibroblasts into a permanent low-contractile and non proinvasive hDF phenotype. While HN-CAF and hDF_LIF treated with one inhibitor initially showed significant reduction of contractile capacity, no significant difference was observed at day 15 compared with the untreated counterparts (Fig. 5b, Supplementary Fig. 5e). On the contrary, on combined treatment, the activated fibroblasts completely and permanently lost all capacity to contract the collagen lattices (50-day assays) that became indistinguishable from the hDF counterparts (Fig. 5b, Supplementary Fig. 5e). Similar abrogative effects were observed after a 21-day treatment (Supplementary Fig. 5f,g). Interestingly, abrogation of the proinvasive phenotype was observed with carcinoma-, LIF- and TGFβ-activated fibroblasts even after removal of the two inhibitors. In cells exposed to only one of the two inhibitors, the abrogative effect was reversed 5 days after the drug removal (Fig. 5c). Also, in an organotypic model of breast carcinoma, Br-CAF promoted collective invasion capacity in the MDA-MB-468 carcinoma cell line that was abrogated by the combined 5′-Aza and Ruxolitinib treatment of the Br-CAF (Fig. 5d). Taken together, these data demonstrate that in hDF, LIF induces an epigenetic-dependent constitutive activation of the JAK1/STAT3 signalling pathway that governs the proinvasive CAF cell ability. Such epigenetic activation is abrogated on the long term by treatments combining JAK1 and DNMT inhibitors. The potential therapeutic effect of targeting the DNMT and JAK pathway during breast carcinomagenesis was evaluated *in vivo*. Thus, mouse dermal fibroblasts (mDF), isolated from back skin of Balb/C mice, were activated *in vitro* by LIF, then submitted to treatment with either one of each inhibitors or a combination of both. Treated mDF were co-implanted in the forth mammary fat pad of BALB/c female mice together with the 67NR mouse mammary carcinoma cell line, that neither invades from the primary tumour, nor activates fibroblasts *in vitro* and *in vivo*^16^ (Fig. 6a). Thirty days after co-implantation, the mice were killed, and the 67NR cell-invasive capacity and tumour ECM remodelling were analysed by immunohistochemistry. 67NR cells, from the primary tumour bulk (T) invaded the adjacent tissue exclusively when co-implanted with LIF-activated mDF, which contrasted with the behaviour of 67NR injected either alone or co-implanted with control mDF (mDF_Veh.). Treatment of mDF_LIF with one inhibitor reduced the onset of a proinvasive microenvironment, while treatment combining both inhibitors restored the non-invasive 67NR cell phenotype (Fig. 6b,c, left panels). The invasive behaviour of 67NR cells correlated with an excessive tumour ECM remodelling disclosed by Picrosirius Red staining and orthogonal light visualization (Fig. 6c, middle and right panels, and Fig. 6d). Taken together, these data indicate that LIF-activated fibroblasts induce onset of a proinvasive tumour microenvironment *in vivo*, in a syngenic and orthotopic mice model of breast cancer.

### STAT3 and SHP-1 inverse expression in human carcinomas

**Figure 7:**
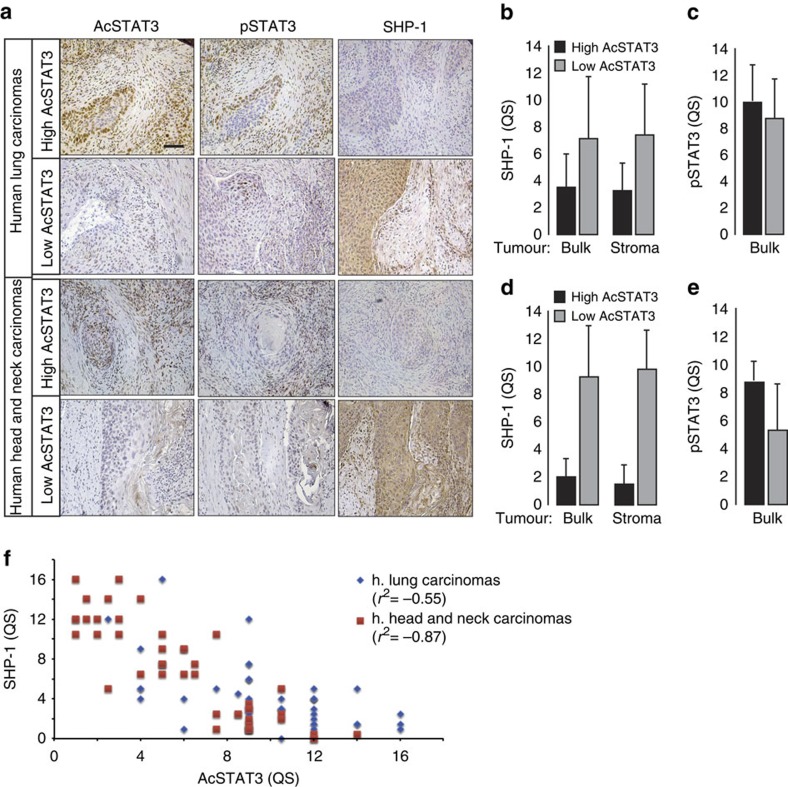
Inverse correlation of AcSTAT3 and SHP-1 expression. (**a**) AcSTAT3, pSTAT3 and SHP-1 immunohistological staining in human head and neck (*n*=50) and lung (*n*=50) carcinomas. Scale bar, 100 μm. (**b**) Quantification of mean Quick Score (QS) for SHP-1, both in the stroma and the tumour bulk, in lung carcinomas decorated with high- and low-acetylated STAT3 shown in **a**. (**c**) Quantification of mean Quick Score (QS) for pSTAT3, in the tumour bulk, in lung carcinomas decorated with high and low acetylated STAT3 shown in **a**. (**d**) Quantification of mean QS for SHP-1, both in the stroma and the tumour bulk, in head and neck carcinomas decorated with high- and low-acetylated STAT3 tumours shown in **a**. (**e**) Quantification of mean Quick Score (QS) for pSTAT3, in the tumour bulk, in head and neck carcinomas decorated with high- and low-acetylated STAT3 shown in **a**. (**f**) Plot of mean QS form AcSTAT3 (*x* axis) and SHP-1 (*y* axis) showing a negative correlation between Acetylated STAT3 and SHP-1 detection in lung (blue) and head and neck (red) carcinomas.

To confirm the *in vitro* findings with *ex vivo* observations, an immunohistological analyses of STAT3 phosphorylation and acetylation together with SHP-1 expression were performed in 100 human carcinoma samples from head and neck (*n*=50) and lung (*n*=50) cancers (Fig. 7a, Supplementary Fig. 6a). The levels of AcSTAT3 detected in lung (Fig. 7b) and head and neck (Fig. 7c) was consistently opposite to SHP-1 expression, which establishes a strong inverse correlation between STAT3-K685 acetylation and SHP-1 expression (*r*^2^=−0.87 for head and neck; *r*^2^=−0.55 for lung carcinomas; Fig. 7d). Thus the *ex vivo* data are consistent with the *in vitro* evidence revealing that STAT3 acetylation leads to SHP-1 downregulation in stromal fibroblasts. No significant difference between phosphorylation and acetylation of STAT3 in the tumour stroma of both head and neck and lung tumours biopsies was observed, which corroborates the *in vitro* evidence that STAT3 acetylation is compulsory for phosphorylation of the STAT3 tyrosine 705 residue (Supplementary Fig. 6a–c). Next, the quick scores obtained from each variable were considered for the design of a predictive model. A principal component analysis was done to highlight dispersed values and build a clean set of measurements avoiding these values. Twenty six values were eliminated from the set. Scatter-plots were made to calculate the correlation matrix for each couple of variables for both the complete and the cleaned set of measurements (Supplementary Table 2A). Correlation coefficients are widely acceptable especially for the cleaned set, suggesting the possibility to define a reliable predictive model. Using linear multivariate logistic regressions three predictive models were thus obtained (Supplementary Table 2B). *In silico* values generated from these models are highly correlated with the *ex vivo* values. Furthermore, the ‘SHP-1∼ac-STAT+p-STAT' model resulted to be relevant and able to predict the SHP-1 level by scoring STAT3 phosphorylation and acetylation only with high level of confidence (Supplementary Fig. 6d).

## Discussion

CAF are responsible for the onset of the proinvasive tumour microenvironment, which favours carcinoma cell dissemination from the primary tumour. Interestingly, the proinvasive properties of CAF remain activated even after their isolation from tumours and propagation *in vitro*^2^,^9^,^10^,^16^,^36^,^37^,^38^. Here, we show that such permanently activated proinvasive status of CAF, isolated from head and neck, lung and breast carcinomas, results from the LIF-mediated persistent activation of the JAK1/STAT3 signalling pathway, which regulates acto-myosin contractility and confers on them the proinvasive capacity of digging tracks within the ECM that are subsequently used by the tumour cells to collectively invade^9^,^15^. Constitutive activation of the JAK1/STAT3 signalling pathway is a process frequently observed in tumour cells^39^,^40^. It involves either genetic mutations activating the GP130-IL6ST receptor^41^, or the JAK2 kinase^42^ or secretion of growth factors and cytokines mediating an autocrine activation of the signalling pathway^43^. While occurrence of oncogenic mutations in CAF is still matter of debate^27^,^44^, in our case, any possible involvement of secreted factors from both CAF and LIF-activated fibroblasts can be excluded (Fig. 1c,d). Activated fibroblasts isolated form tumours or fibrotic tissues present a globally hypomethylated DNA profile that strikingly contrasts with a hypermethylated pattern of the promoters of tumour-suppressor genes such as *Thy1, tgfbr2* or *PTEN*^24^,^45^,^46^,^47^,^48^,^49^,^50^. Moreover, epigenetic modifications are considered a crucial step in the constitutive fibroblast activation in a mouse model of kidney fibrosis^12^. Consistent with such observations, we have identified the DNMT family epigenetic modifiers as regulators of the proinvasive CAF activity that acts on the JAK1 and STAT3 constitutive activation. In this context, we provide evidence that LIF induces an initiation phase that requires DNMT3b transcriptional expression and its association with AcSTAT3 to promote methylation of the *PTPN6* tyrosine phosphatase promoter. Subsequently, the DNMT1-dependent replication methylation of the *PTPN6* promoter maintains silent the SHP-1 expression, which causes constitutive tyrosine phosphorylation of JAK1 resulting in fibroblast activation. Epigenetic regulation of the JAK1/STAT3 pathway has been suggested in tumour cells^19^,^22^,^51^,^52^. However, we demonstrate for the first time that in cancer epigenetic downregulation of SHP-1 sustains the proinvasive activity of the stromal fibroblasts. Interestingly, this observation is corroborated by the strong inverse correlation existing between detection of acetylated STAT3 and expression of SHP-1 observed in human tissues obtained form head and neck and lung tumour specimens. In light of our results, we propose that *PTPN6*, a tumour-suppressor gene in leukaemia and lymphoma, acts as a fibrotic suppressor gene in CAF from head and neck, lung and breast cancers.

We further disclose a crucial role for p300 HAT in proinvasive HN-CAF phenotype (Fig. 2). Indeed, the transcriptional co-activator p300 has been implicated in diverse biological functions, including tissue fibrosis^53^, and its catalytic acetyltransferase activity is enhanced in fibroblasts in response to TGFβ (ref. 44) and in tumour cells by the LIF or IL6 cytokines^54^. In our system, specific inhibition of p300 abolishes the LIF- and carcinoma-associated proinvasive fibroblast activity, suggesting that, likewise in several human diseases^55^, in stromal fibroblasts p300 and its homologue CBP possess distinct functions. Experimental evidences attribute a clear role for both p300 and CBP in tumorigenesis as tumour-suppressor genes in haematological tumours and through regulation of tumour suppressing signalling pathways, such as TGFβ and p53, in cancer cells^56^. Our results disclose a procarcinogenic function for p300. Indeed, p300 activation by inflammatory cytokines leads to permanent JAK1/STAT3 signalling activation through the DNMT3b-dependent SHP-1 tyrosine phosphatase silencing and proinvasive stromal fibroblast initiation.

DNA epigenetic modification is a reversible process. Reversion can be induced by treatments with the 5′-Aza that degrades DNMT^57^. However, in patients with myelodisplastic syndromes, re-expression of tumour-suppressor genes consequent to treatment with 5′-Aza or decitabine is transient and concomitant with reversion of the methylation profiles^58^. In our system, a 7-day treatment on both HN- and Br-CAF or hDF_LIF combining both 5′-Aza and the JAK1/2 inhibitor Ruxolitinib reverts the proinvasive properties of activated fibroblasts on the long term. Association of both compounds is mandatory for such a long-term effect, because treatments using each single-chemical compounds only transiently inhibit the CAF-dependent matrix remodelling, SCC12 cells invasion and SHP-1 protein expression. It is conceivable that the passive DNA demethylation induced by 5′-azacytidine^31^ is not sufficient to permanently revert the CAF phenotype, whereas active genomic demethylation, through replacement of modified cytosine in the absence of cell division^59^ may explain such a prolonged effect of the combined inhibitor treatment. Indeed, BMP7-induced inhibition of kidney fibrosis is mediated by an active genomic Tet3-dependent *Rasal1* promoter demethylation^60^. Hesson *et al*.^61^, suggested that nucleosome reassembly is a crucial step that dictates the transient efficacy of DNMT inhibitors. We therefore hypothesize that inhibition of DNMT activity, together with JAK inhibition may lead to histone modification, such as acetylation and methylation, resulting in the inhibition of nucleosome reassembly within the *SHP-1* promoter.

In conclusion, our work establishes that acquisition and long-term maintenance of head and neck, lung or breast CAF proinvasive ability relies on a two-step mechanism. In hDF, LIF initiates this process through STAT3 acetylation by p300 and DNMT3b-dependent methylation of the *PTPN6* promoter, which is followed by a long-term maintenance phase mediated by a DNMT1-dependent loss of SHP-1 expression and JAK1 kinase constitutive activity. In light of our results obtained by combining the use of 5′-Aza and Ruxolitinb, both drugs approved by the Food and Drug administration, we demonstrate phenotypical and molecular reversion of activated fibroblasts into ‘normal' fibroblast-like cells and we propose that such treatment might represent an attractive approach to inhibit the onset of the proinvasive tumour microenvironment in cancer.

## Methods

### Cell culture

Human primary dermal fibroblasts (hDF), MDA-MB-468 and human HEK293 Phoenix cells were maintained in DMEM supplemented with 10% FCS (fetal calf serum). Human CAF isolated from patients with head and neck, lung and breast cancers were cultured in DMEM supplemented with 10% FCS and insulin-transferrin-selenium (#41400-045; Invitrogen, Carlsbad, CA). SCC12 cells were cultured in FAD media, as described in Gaggioli *et al*.^9^.

Long-term LIF and TGFβ1-activated fibroblasts (hDF_LIF and hDF_TGFβ1) have been performed in DMEM supplemented with 0.5% FCS containing 2 ng ml^−1^ final concentration of human recombinant proteins for 7 days. Next, activated-hDF was cultured for 15 days (otherwise stated) in 0.5% FCS media before experiments (as described in Supplementary Fig. 1a).

### Cytokines and neutralizing antibodies and inhibitors

TGFβ1 was purchased from Peprotech (#100-21, Peprotech, Rocky Hill, NJ) and was used at 2 ng ml^−1^; recombinant human LIF was purchased from Millipore (#LIF1005,Millipore, Billerica, MA), and was used at a concentration of 2 ng ml^−1^. LIF neutralizing antibody (AB-250-NA, R&D, Minneapolis, MN) was used at 10 μg ml^−1^. Neutralizing antibodies were incubated for 1 h with CM before experiments. The following inhibitors were used in this study: Pyridone 6 (#42009, Calbiochem, Los Angeles, CA) was used at 5 μM, Ruxolitinib (#1598, Axon medchem, Groningen, The Netherlands) at 10 μM, 5′-Aza (#A2385, Sigma, Saint Louis, USA) was used daily at 2.5 μM, PTP inh 1 (#540200, Millipore, Billerica, MA) at 10 μM, Sodium Orthovanadate (#567540, Millipore, Billerica, MA) at 1 mM, C646 (#SML002, Sigma, Saint Louis, USA) and CTPB (#sc- 202558, Santa Cruz, Biotechnology, Santa Cruz, CA) at 25 μM. A list of the concentrations used for the inhibitors/activators in Fig. 1d,e is provided in Supplementary Table 1.

### RNAi transfections

Cells were plated at 60% confluence and subjected to transfection the following day using Dharmafect 3 (#T-2002-02; Dharmacon, Inc., Lafayette, CO) at 100 nM final concentration of RNAi. For long-term activation studies, fibroblasts were transfected once at day 0, stimulated with recombinant cytokines from day 1 to day 7, retransfected at day 8, and maintained in 0.5% medium until day 15 for extracellular matrix remodelling, organotypic invasion assays and protein extraction. RNAi sequences are listed below:

p300 #1—5′-GGACUACCCUAUCAAGUAA-3′

p300 #2—5′-GACAAGGGAUAAUGCCUAA-3′

HDAC3 #1—5′-AACAAGAUCUGUGAUAUUG-3′

HDAC3 #2—5′-GGAAUGCGUUGAAUAUGUC-3′

HDAC3 #3—5′-GCACCCGCAUCGAGAAUCA-3′

HDAC3 #4—5′-AAAGCGAUGUGGAGAUUUA-3′

DNMT1 #1—5′-GCACCUCAUUUGCCGAAUA-3′

DNMT1 #2—5′-AUAAAUGAAUGGUGGAUCA-3′

DNMT1 #3—5′-CCUGAGCCCUACCGAAUUG-3′

DNMT1 #4—5′-CGACGACCCUGACCUCAAA-3′

DNMT3b #1—5′-ACGCACAGCUGACGACUCA-3′

DNMT3b #2—5′-UUUACCACCUGCUGAAUUA-3′

DNMT3b #3—5′-CGAAAUAACAACAGUGUCU-3′

DNMT3b #4—5′-GCUCUUACCUUACCAUCGA-3′.

### Organotypic invasion assays and matrix remodelling assays

For Organotypic invasion assays, 5 × 10^5^ fibroblasts were embedded in 1-ml matrix gel. After 1 h at 37 °C, matrix gel were overlaid with 5 × 10^5^ SCC12 cells and lifted at the cell-air interface 24 h later for 5 days, the cultures were then fixed, embedded in paraffin blocks, sectioned and stained for invasion index quantification using ImageJ^30^. For gel contraction assay, 25 × 10^3^ cells were embedded in 100 μl of matrix gel and seeded in triplicate into 96-wells plate. After 1 h at 37 °C, matrix gels were overlaid with 100 μl of 0.5% FCS medium (with indicated cytokines or inhibitors) and changed every 2 days. At day 6, the relative diameter of the well and the gel were measured using ImageJ. The percentage of gel contraction was calculated using the formula 100 × (well diameter−gel diameter)/well diameter.

### Antibodies

Antibodies against STAT3 (#9139; 1/1,000), pY705-STAT3 (#9145; 1/1,000), ac-K685 STAT3 (#2523; 1/1,000), SMAD2 (#3122; 1/1,000), pSer465/467-SMAD2 (#3108; 1/1,000), pY1022/1023-JAK1 (#3331; 1/200), JAK1 (#3332; 1/500), MLC2 (#3672; 1/500) and pThr18/19-MLC2 (#3674; 1/500) were purchased from Cell Signalling (Cell SignalingTechnology, Beverly MA), α-tubulin from sigma (T4026, Sigma, Saint Louis, MO; 1/5,000); SHP-1 (#sc-7289, 1/ 500 for western blot analysis), DNMT1 (#sc-20701; 1/1,000), DNMT3b (#sc-130740; 1/1,000) from Santa Cruz Biotechnology (Santa Cruz Biotechnology, Santa Cruz, CA) and SHP-1 (#ab2020; 1/200 for immunohistochemical staining) from Abcam (#ab2020, Abcam, Cambridge, UK) from Bethyl Laboratories (Bethyl Laboratories, Montgomery, TX, USA).

### Western blots and Co-immunoprecipitation analysis

For immunoblotting analysis, cells were lysed on ice in lysis buffer (25 mM Tris (pH 6,8), 2% SDS, 5%glycerol, 1% β-mercaptoethanol, 0.01% bromophenol blue). Equal amounts of protein from each sample were loaded on SDS–PAGE, separated, and transferred onto nitrocellulose. The immunoblots were blocked by incubation in 5% bovine serum albumin, 10 mM Tris-HCl (pH 7.5), 500 mM NaCl, 0,1% Tween 20 for 30 min at room temperature, probed with specific antibodies and then with secondary antibodies using common classical methods. Immunodetection was performed using chemiluminescent HRP substrate (#WBKLS0500, Millipore, Billerica, MA). Uncropped western blots are shown in the Supplementary Information section and labelled as Supplementary Fig. 7.

For co-immunoprecipitation analysis, cells were lysed on ice in modified RIPA buffer (50 mM Tris pH 7.4 150 mM NaCl, 1%NP-40, 0.1% SDS, 0.5% SD Dexoxycholate, 5 mM NaF, 2.5 mM Nappi, and protease inhibitor (#04693159001, Roche)) for 30 min and isolated by centrifugation (30 min, 10,000 *g*, 4 °C). Supernatants were precleared with Magna CHIP protein G bead (#16-662, Millipore) and the cleared lysate incubated with primary antibody overnight at 4 °C. Immune complexes were captured by adding 35 μl of protein G magnetic beads, rotated for 1 h at 4 °C and washed three times with lysis buffer (without SDS, sodium deoxycholate and protease inhibitors). Immunoprecipitation products were separated by SDS–PAGE.

### RT–qPCR analysis

RNA isolation was performed using RNeasy Mini kit (#217004, Qiagen, Turnberry Ln, Valencia, CA) according to the manufacturer's instructions. Reverse transcription (RT) of 500 ng RNA by Superscript II reverse transcriptase (#18064-014, Invitrogen, Carlsbad, CA) was followed by real-time PCR using Fast SYBR Green Master Mix (#18064-014; Applied Biosystems, Foster City, CA) and performed on a Step One Plus Real-Time PCR system (Applied Biosystems, Foster City, CA).

Primers sequences are listed as follows:

DNMT1: sense, 5′-CCAAAGCCCGAGAGAGTGCCTCAG-3′ and antisense, 5′-CCTTAGCAGCTTCCTCCTCCTT-3′;

DNMT3a: sense, 5′-CTGAAGGACTTGGGCATTCAG-3′ and antisense, 5′-CACCATGCCCACCGTGA-3′;

DNMT3b: sense, 5′-TACACAGACGTGTCCAACATGGGC-3′ and antisense, 5′-GGA-TGC-CTTCAGGAATCACACCTC-3′;

GAPDH: sense, 5′-ACCCAGAAGACTGTGGATGG-3′, and antisense, 5′-TCTAGACGGCAGGTCAGGTC-3′.

Relative expression of the respective gene was determined after normalization to GAPDH and calculated with the following formula: relative expression level=2ddCT.

### Statistical analysis

Student's *t*-test was performed for statistical analysis of invasion assay, gel contraction assay, and quantitative PCR (qPCR) results. ***P<0.001, **P<0.01 and *P<0.05. Error bars are +s.d. Pearson's correlation coefficient was used to assess the relationship between pSTAT3, AcSTAT3 and SHP-1 quick score within human samples.

### *In silico* linear multivariate logistic regression

Quick score experimental values from 100 carcinomas were considered to design a model allowing predicative values depending of 2 others. A principal component analysis was done to highlight dispersed measurements and build a clean set (*n*=74) suitable for building the model. Correlations between the variables were checked and a linear multivariate logistic regression achieving to determine the model. Statistical analyses were performed using R software^62^.

### Immunohistochemical staining and quantification methods

Fifty head and neck and 50 lung tumour biopsies were fixed (3.7% formaldehyde in PBS) for 4 h and transferred to 70% ethanol (24 h), embedded in paraffin wax and sectioned at 7 μm. After deparaffination, microwave antigen retrieval was performed in Na-citrate buffer (10 mM, pH6; 5 min at 900 W, 10 min at 150 W and 30 min at room temperature) for SHP-1 staining, and in EDTA buffer (1 mM, PH8, 5 min at 900 W and 25 min at 150 W) for DNMT3b, pSTAT3 and AcSTAT3 staining. Sections were washed three times in PBS (5 min per wash) before endogenous peroxidase activity was blocked in 1% H2O2 for 10 min and washed three times in PBS. After incubation in blocking buffer for 2 h (10% serum (S-5000, S-1000; Vector, Burlingame, CA); 0.3% Triton X100 in PBS), sections were incubated with primary antibody diluted 1:50 in blocking buffer overnight at 4 °C. After three washes in PBS, sections were incubated with biotinylated secondary antibody (#BA-1000 Vector, Burlingame, CA) diluted 1:400 in PBS for 30 min and washed three times in PBS. Samples were then processed using Vectastain ABC kit (#PK4001, Vector, Burlingame, CA) and DAB peroxidase substrate kit (#SK4100, Vector, Burlingame, CA) according to manufacturer's instructions. Sections were next counterstained with haematoxylin for 5 s, rinsed in water, blued 10 s in 0,08% ammonia water, dehydrated, cleared and mounted with cover clips. Two authors, blinded to each other's assessment, scored the slides using the Quick Score method^16^ to determine pSTAT3, AcSTAT3, DNMT3b and SHP-1 status within the tumour bulk and stroma. The protocol was approved by the local ethic committee of the Nice University Hospital. All patients signed an informed consent for inclusion into the research project.

### Picrosirius Red stain and quantification

Picrosirius Red stain was achieved through the use of 5-mm paraffin sections stained with 0.1% Picrosirius Red (Direct Red80, Sigma-Aldrich) and counterstained with Weigert's haematoxylin to reveal fibrillar collagen. The sections were then serially imaged using with an analyser- and polarizer-oriented parallel and orthogonal to each other. Microscope conditions (lamp brightness, condenser opening, objective, zoom, exposure time and gain parameters) were constant throughout the imaging of all samples. A minimal threshold was set on appropriate control sections for each experiment in which only the light passing through the orthogonally oriented polarizers representing fibrous structures (that is, excluding residual light from the black background) was included. The threshold was maintained for all images across all conditions within each experiment. The area of the transferred regions that was covered by the thresholded light was calculated and at least five × 20 field per condition were averaged together (Image J software).

### Orthotopic syngenic tumours xenograft in BALB/c mice

Eight-week-old female BALB/c mice were anaesthetized using ketamine and xylazine by peritoneal injection. Skin was incised and 10^6^ 67NR were co-injected into the right fourth mammary fat pad in 10 μl PBS with or without 10^6^ BALB/c dermal fibroblast (isolated from the back skin of 6- to 10-week-old BALB/c mice) treated as described in Fig. 6a. Mice were killed 30 days post injection, primary tumours were removed. After excision and 12 h of fixation in 3.7% neutral-buffered formalin at 25 °C, tumours were paraffin-embedded via an ethanol–xylene dehydration series, before being sliced into 5-μm sections and stained with haematoxylin and eosin or Picrosirius Red. The protocol was approved by the local committee of the host institute and by the Institutional Animal Care and Use Committee (CIEPAL AZUR committee, MESR number 2015051917125051) at the University of Nice-Sophia Antipolis, Nice, France.

### DNMT activity assay

Episeeker DNMT activity quantification kit (#ab113467, Abcam, Cambridge, UK) was used to assess DNMT activity on 10 μg of nuclear proteins (nuclear extracts were prepared using the EpiSeeker Nuclear Extraction Kit (#ab113474, Abcam, Cambridge, UK)) following manufacturer's specifications.

### Plasmids constructs

pcDNA5-mSTAT3-K685R (in which K685 is mutated from AAG (K) to AGG (R)) was derived from the wild-type pCDNA5-mSTAT3-GFP-FRT expression vector (which was a gift from the laboratory of Dr. Müller-Newen) using the Quick Change II site-directed mutagenesis kit (Agilent Technologies, Santa Clara, CA, USA). The sequence of the oligonucleotide primers used are: STAT3-K685R-Forward: 5′-GAGGAGGCATTTGGAAGGTACTGTAGGCCCGAG-3′; and STAT3 K685R-Reverse: 5′-CTCGGGCCTACAGTACCTTCCAAATGCCTCCTC-3′ and constructs were then coupled to GFP. Following constructs verification by nucleotide sequencing, the STAT3 (wild-type and K685R) fragments were excised from pcDNA5 by Pme1 and BamH1 digestion and directionally cloned into pBabe retroviruses expression vectors (containing the puromycin resistance gene as a selectable marker) digested with NaeI and BamH1.

SHP-1 cDNA was isolated from hPF by RT–PCR and subcloned into a pBabe retrovirus expression vector.

### Production of recombinant retroviruses

Phoenix cells were transiently transfected with 1 μg of the previously described vectors using calcium phosphate mediated transfection using classical procedures. Six hours after transfection, cells were washed with PBS and complete media was added. The day after, media was replaced by a heat-inactivated serum medium and cells were moved to 32 °C for 24 h. Forty-eight hours post transfection clarified supernatents (retroviral particles) were collected and used to infect either hDF or CAF. Retrovirus infection was performed in the presence of 5 μg ml^−1^ polybrene. Stably transduced cells were selected with 5 μg ml^−1^ puromycin.

### Bisulfite sequencing

Genomic DNA was extracted with the QIAamp DNA Blood Mini Kit (Qiagen). Bisulfite sequencing was conducted with the EZ DNA Methylation Start-up kit (Zymo Research). Briefly, 200 ng of genomic DNA in CT Conversion Reagent buffer was denatured for 8 min at 98 °C and incubated for 3.5 h at 64 °C. After on-column desulfonation, DNA was eluted in 10 μl elution buffer, and subjected to 40 cycles of PCR amplification (95 °C for 30 s, 59 °C for 30 s, 72 °C for 1 min) following an initial denaturation step at 95 °C for 10 min. Primers in the SHP-1 (PTPN6) promoter (LOU1398, LOU1399, LOU1400 and LOU1401) were designed using the MethPrimer online software (www.urogene.org/methprimer/index1.html). Their specificity for bisulfite converted DNA was verified by PCR amplification of bisulfite-treated and -untreated genomic DNA. PCR products were gel-purified and cloned into pGEM-T (pGEM-T Easy Vector System, Promega). Transformants containing recombinant plasmids were selected by blue/white colony screening and randomly picked up for Sanger sequencing. Analysis was performed using the QUMA online software^63^ with the default conditions (exclusion of sequences with a bisulfite conversion rate below 95% or with an identity to the reference sequence lower than 90%). Statistical analysis was performed using the GraphPad Prism 6 software.

Oligos:

Region 1, fwd: LOU1398

5′-GGTTTGTATAGGGGGTATTTTTTTT-3′

Region 1, rev: LOU1399

5′-CACCTAAAACTCCACCTAAAACTCA-3′

Region 2, fwd: LOU1400

5′-AGTTTTAGGTGGAGTTTTAGGTGGT-3′

Region 2, rev: LOU1401

5′-CAAAAACAAACCCTAAACTAATAATCTC-3′.

## Additional information

**How to cite this article:** Albrengues, J. *et al*. Epigenetic switch drives the conversion of fibroblasts into proinvasive cancer-associated fibroblasts. *Nat. Commun.* 6:10204 doi: 10.1038/ncomms10204 (2015).

## Supplementary Material

Supplementary Information Supplementary Figures 1-7 and Supplementary Tables 1-2
