# Supplementary material for: Epigenetic switch drives the conversion of fibroblasts into proinvasive cancer-associated fibroblasts
Source: Nat Commun. 2015 Dec 15;6:10204. doi: 10.1038/ncomms10204 (PMC4682161; doi:10.1038/ncomms10204)
Supplement: Supplementary Information — Supplementary Figures 1-7 and Supplementary Tables 1-2 [file ncomms10204-s1.pdf]

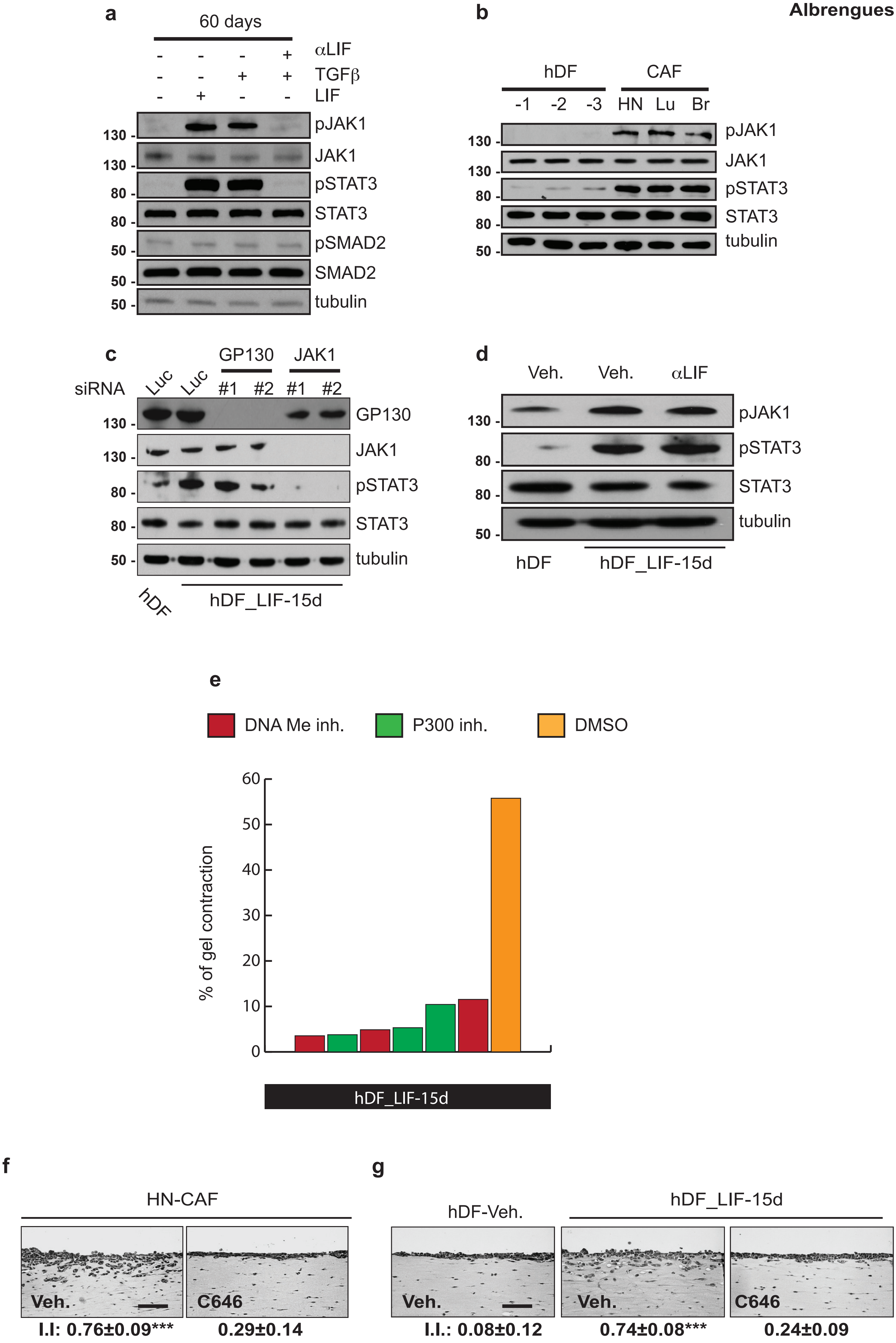

Supplementary Figure 1

Supplementary Figure 1

a) Immunoblot of pJAK1, pSTAT3 and pSmad2 of 7 days LIF- and TGFβ1-activated hDF followed by long-term (60 days) cultured in low serum. Immunoblot of JAK1, STAT3, Smad2 and tubulin as control. b) Immunoblot of pJAK1 and pSTAT3 in three hDF (-1, -2 and -3) isolated from normal skin and three CAF (HN, Lu and Br). Immunoblot of JAK1, STAT3 and tubuline as control. c) Immunoblot of GP130 and pSTAT3 in hDF and hDF_LIF transfected with RNAi control (siLuc) or targeting GP130 (siGP130#1 and #2) or JAK1 (siJAK1#1 and #2). Immunoblot of JAK1, STAT3 and tubulin as control. d) Immunoblot of pJAK1 and pSTAT3 in hDF and hDF_LIF in presence of IgG control (Veh.) or LIF blocking antibody (αLIF). Immunoblot of STAT3 and tubulin as control. e) Quantification of matrix remodeling by hDF-LIF in presence of small molecules inhibitors for 6 days. (n=2 in triplicates). f) Representative images of H&E coloration of paraffin-embedded sections of SCC12 in response to control hDF (Veh.) or C646 inhibitor. Scale bar 100µm (n=3; mean ±SD; ***p<0.001). g) Representative images of H&E coloration of paraffin-embedded sections of SCC12 in response to hDF control (Veh) or hDF-LIF in presence or absence of C646 inhibitor. Scale bar 100µm (n=3; mean ±SD; ***p<0.001).

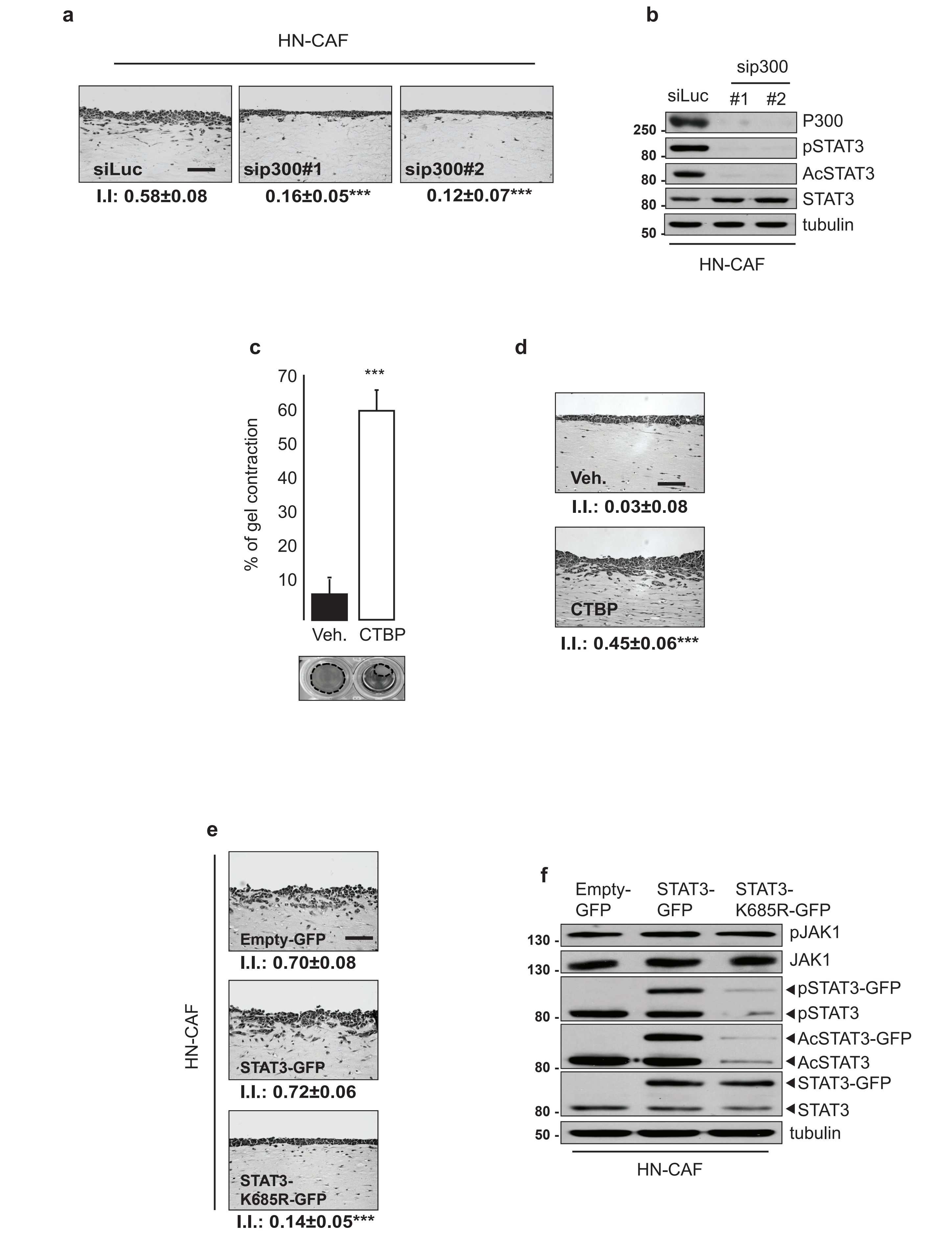

Supplementary Figure 2

Supplementary Figure 2

a) Representative images of H&E coloration of paraffin-embedded sections of SCC12 in response to CAF transfected with control siRNA control (siLuc) or targeting p300 (sip300#1 and #2). Scale bar 100µm (n=3; mean ±SD; ***p<0.001). b) Immunoblot of p300, pSTAT3 and AcSTAT3 in CAF transfected with RNAi control (siLuc) or targeting p300 (sip300#1 and #2). Immunoblot of STAT3 and tubulin as control. c) Percentage of gel contraction by hDF cells in absence (Veh.) or presence of CTPB small molecule activator (n=3 in triplicate, mean + SD, ***p<0.001). d) Representative images of H&E coloration of paraffin-embedded sections of SCC12 in response to hDF cells in absence (Veh.) or presence of CTPB small molecule activator (n=3, mean ± SD, ***p<0.001). e) Representative images of H&E coloration of paraffin-embedded sections of SCC12 in response to CAF overexpressing a control (empty-GFP) or a wilt type STAT3 (STAT3-GFP) or acetylated-deficient STAT3 mutant (STAT3-K685R-GFP). Scale bar 100µm (I.I. = Invasion index; n=3; mean ±SD; ***p<0.001). f) immunoblot of pJAK1, pSTAT3 and AcSTAT3 in CAF overexpressing a control vector (empty-GFP), a WT STAT3 (STAT3-GFP) or acetylated-deficient STAT3 mutant (STAT3-K685R-GFP). Immunoblot of JAK1, STAT3, and tubulin as control.

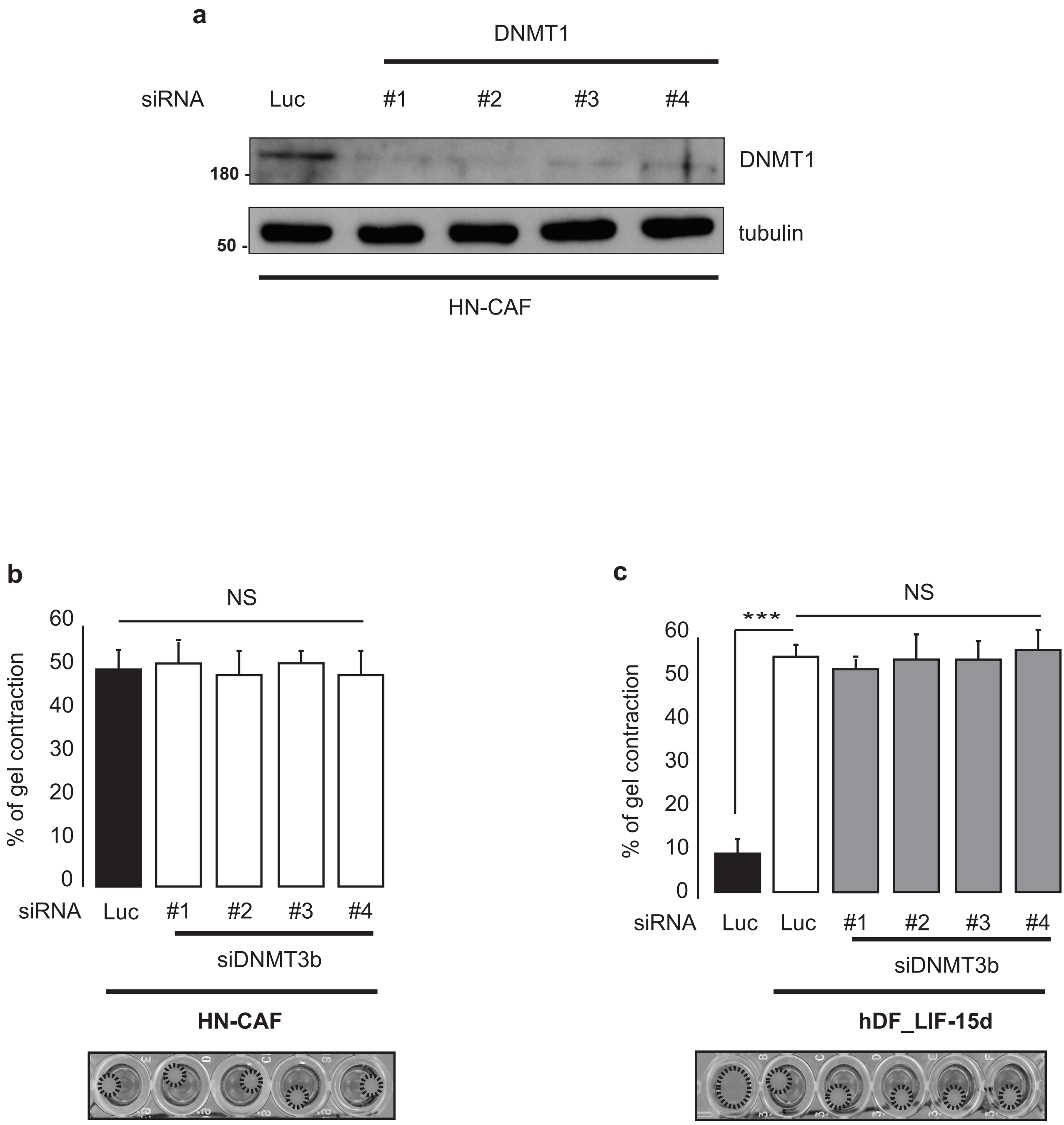

Supplementary Figure 3

Supplementary Figure 3

a) Immunoblot of DNMT1 in CAF transfected with siRNA control (siLuc) or targeting DNMT1 (siDNMT1#1, #2, #3, #4). Immunoblot of tubulin as control. b) Percentage of gel contraction by CAF transfected by RNAi control (siLuc) or targeting DNMT3b (siDNMT3b#1, #2, #3 and #4). Bottom panel show image of the contracted gel (n=3 in triplicate, mean + SD, ***p<0.001). c) Percentage of gel contraction by control hDF and long-term LIF-activated hDF transfected by RNAi control (siLuc) or targeting DNMT3b (siDNMT3b#1, #2, #3 and #4). Bottom panel show image of the contracted gel (n=3 in triplicate, mean + SD, ***p<0.001).

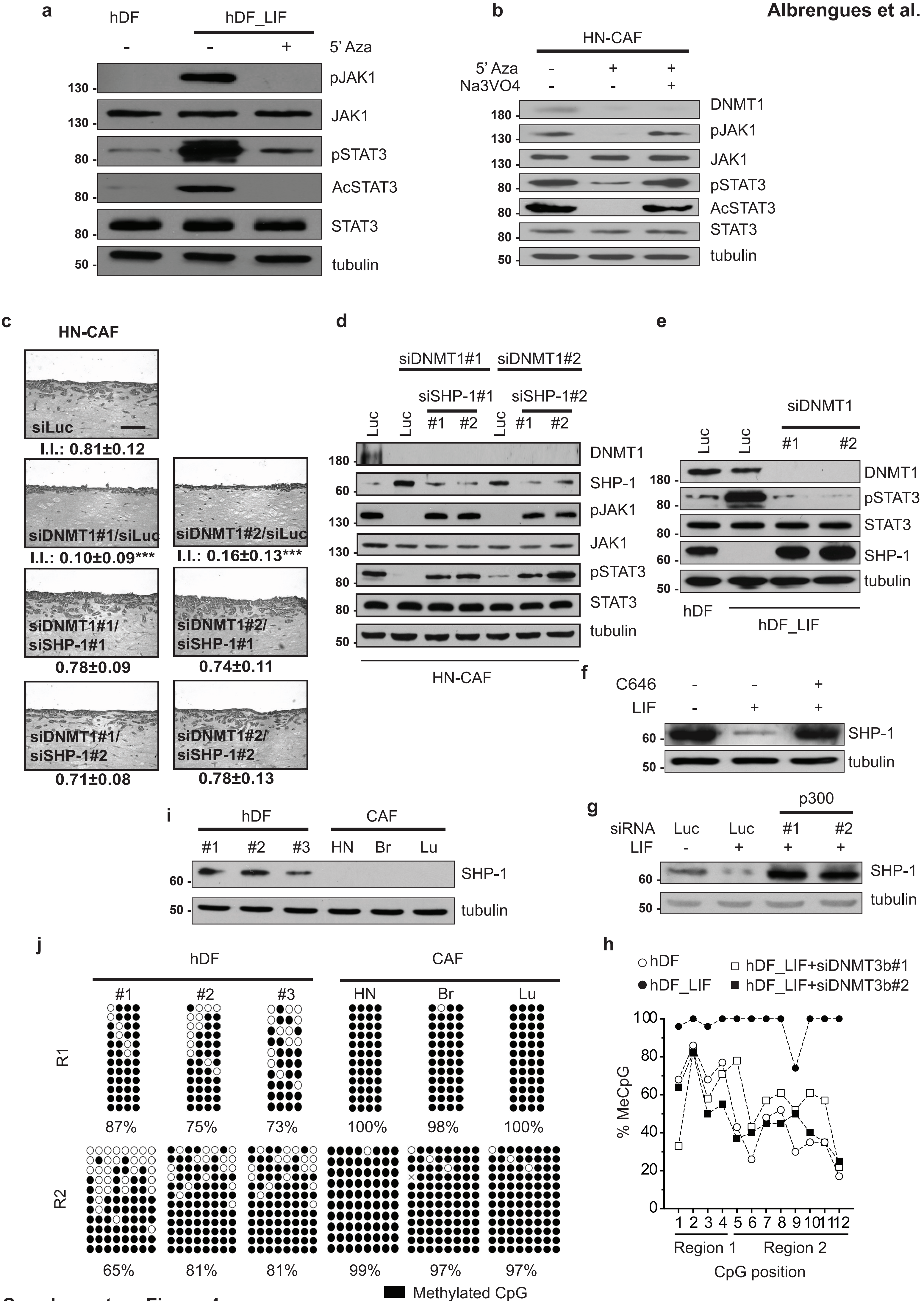

Supplementary Figure 4

Supplementary Figure 4

a) Immunblot of pJAK1, pSTAT3 and AcSTAT3 in hDF stimulated or not by LIF in presence or absence of 5'azacytidine (5' Aza) inhibitor at 10μM. Immunoblot of JAK1, STAT3 and tubulin as control. b) Immunoblot of DNMT1, pJAK1, pSTAT3 and AcSTAT3 in CAF in absence or presence of 5' Aza and Orthovanadate (Na3Vo4) inhibitors. Immunoblot of JAK1, STAT3 and tubulin as control. c) Representative images of H&E coloration of paraffin-embedded sections of SCC12 in response to CAF transfected with siRNA control (siLUC) or with siRNA targeting DNMT1 alone (siDNMT1#1, #2) or in combinaision with siRNA targeting SHP-1 (siSHP-1#1, #2) (n=3, mean ± SD, ***$p<0.001$). d) Immunoblot of DNMT1, SHP-1, pJAk1, pSTAT3 and AcSTAT3 in CAF transfected with siRNA control (siLUC) or with siRNA targeting DNMT1 alone (siDNMT1#1, #2) or in combinaision with siRNA targeting SHP-1 (siSHP-1#1, #2). Immunoblot of JAK1, STAT3 and tubulin as control. e) Immunoblot of DNMT1, pSTAT3 and SHP-1 in hDF and hDF_LIF transfected with siRNA control (Luc) or targeting DNMT1 (DNMT1#1 and #2). Immunoblot of STAT3 and tubulin as control. f) Immunoblot of SHP-1 in hDF treated or not with C646 prior to control or LIF stimulation. Immunoblot of tubulin as control. g) Immunoblot of SHP-1 in hDF transfected with siRNA control (siLUC) or with siRNA targeting p300 (sip300#1, #2) prior to control or LIF stimulation. Immunoblot of tubulin as control. h) Global *PTPN6* promoter methylation analysis in hDF treated with LIF and siRNA against DNMT3b. The graph shows the percentage of methylation for each of the 20 distinct CpG dinucleotides in Region 1 and 2 (numbered from 1 to 20). i) Immunoblot of SHP-1 in three hDF and three CAF. Immunoblot of tubulin as control. j) Bisulfite sequencing results in two regions of the PTPN6 promoter (R1 and R2) in three hDF and three CAF. Each line represents an individual sequence. Open and closed circles denote

unmethylated and methylated CpG dinucleotides, respectively. Global proportion of methylated CpG with the percentage is shown.

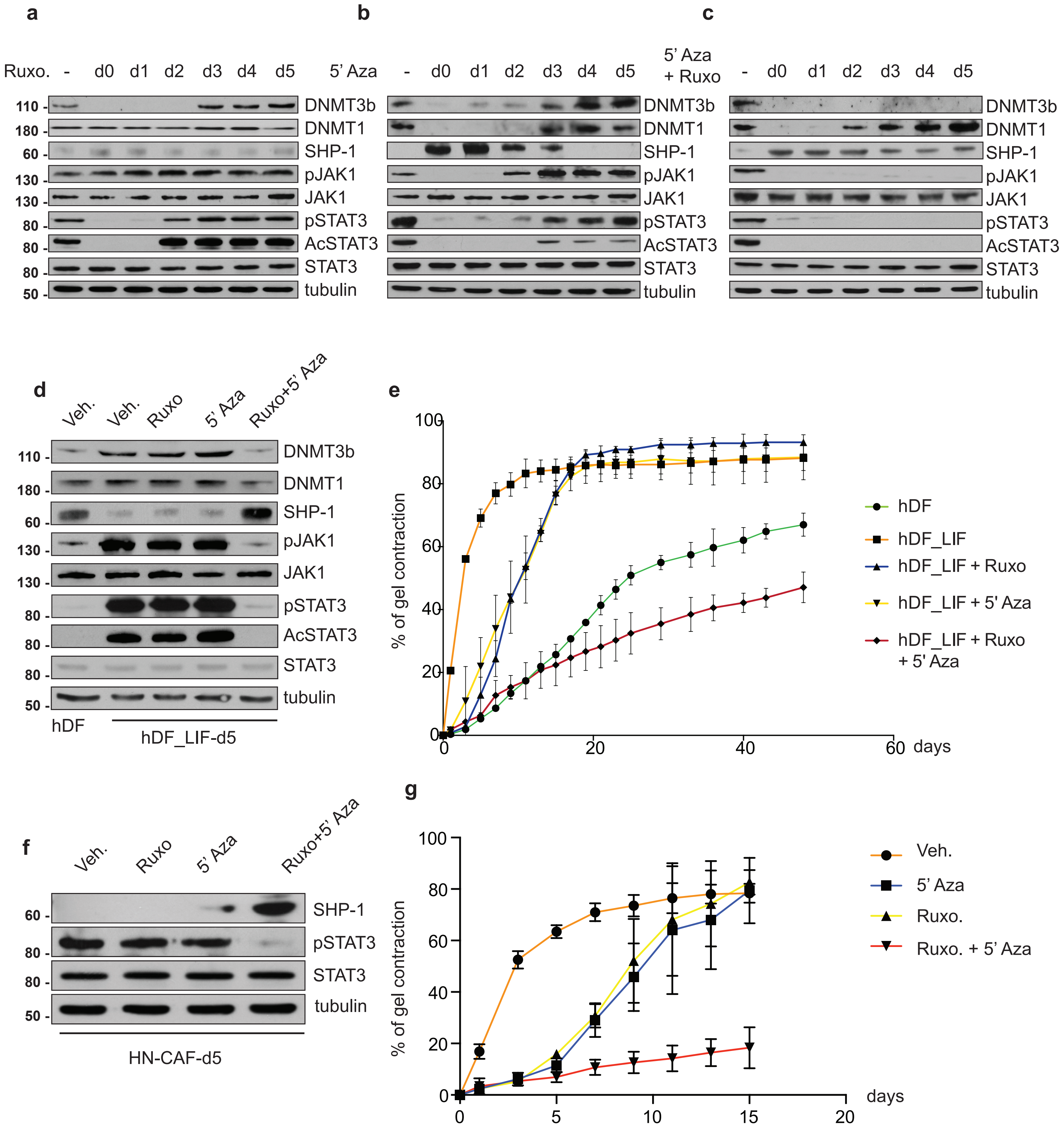

Supplementary Figure 5

Supplementary Figure 5

a) Immunoblot of DNMT3b, DNMT1, SHP-1, pJAk1, pSTAT3, and AcSTAT3 in CAF for 5 days following Ruxolitinib inhibitor treatment. Immunoblot of JAK1, STAT3 and tubulin as control. b) Immunoblot of DNMT3b, DNMT1, SHP-1, pJAk1, pSTAT3, and AcSTAT3 in CAF for 5 days following 5’ Aza inhibitor treatment. Immunoblot of JAK1, STAT3 and tubulin as control. c) Immunoblot of DNMT3b, DNMT1, SHP-1, pJAK1, pSTAT3, and AcSTAT3 in CAF for 5 days following Ruxolitinib and 5’ Aza inhibitors treatment. Immunoblot of JAK1, STAT3 and tubulin as control. d) Immunobot of DNMT3b, DNMT1, SHP-1, pJAK1, pSTAT3 and AcSTAT3 of hDF_LIF in presence for seven days of Ruxolitinib, 5’ Aza or combination of both at day 5 after inhibitors removal. Immunoblot of JAK1, STAT3 and tubulin as control. e) Percentage of gel contraction of hDF control and hDF_LIF for a period of 50 days following seven days of Ruxolitinib, 5’ Aza or both inhibitors treatment. f) Immunobot of SHP-1 and pSTAT3 of CAF in presence for 21 days of Ruxolitinib, 5’ Aza or combination of both at day 5 after inhibitors removal. Immunoblot of JAK1, STAT3 and tubulin as control. g) Percentage of gel contraction of CAF for a period of 15 days following 21 days of Ruxolitinib, 5’ Aza or both inhibitors treatment.

a

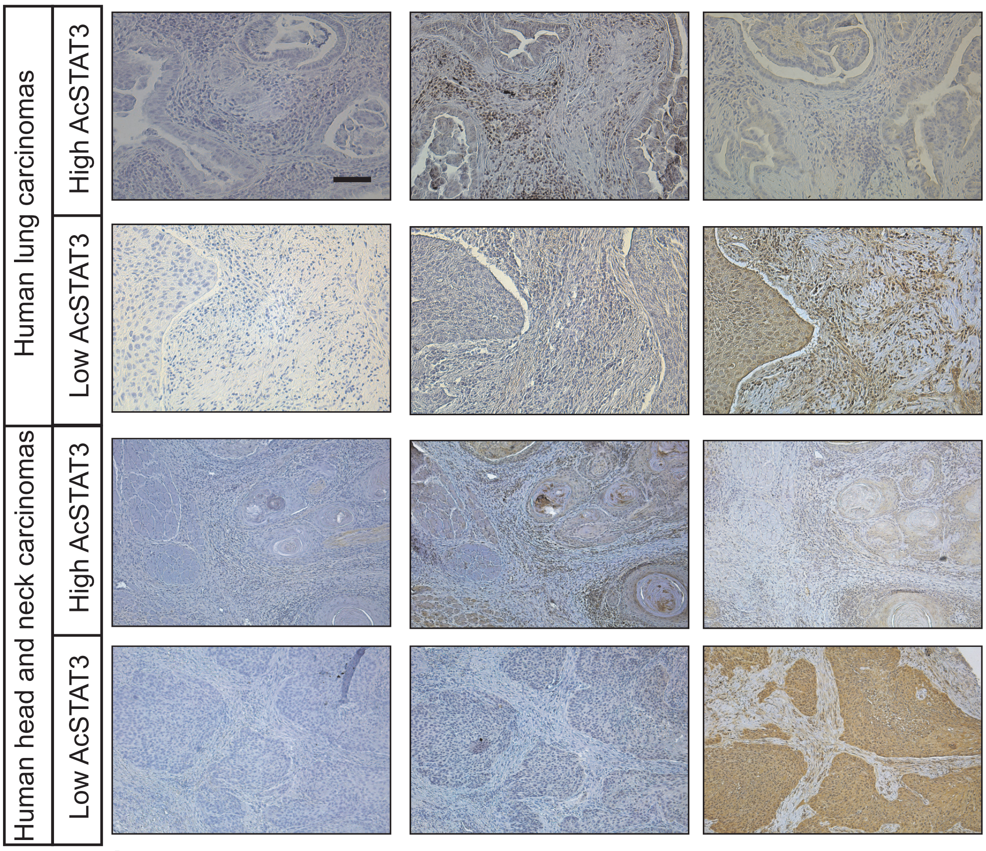

b

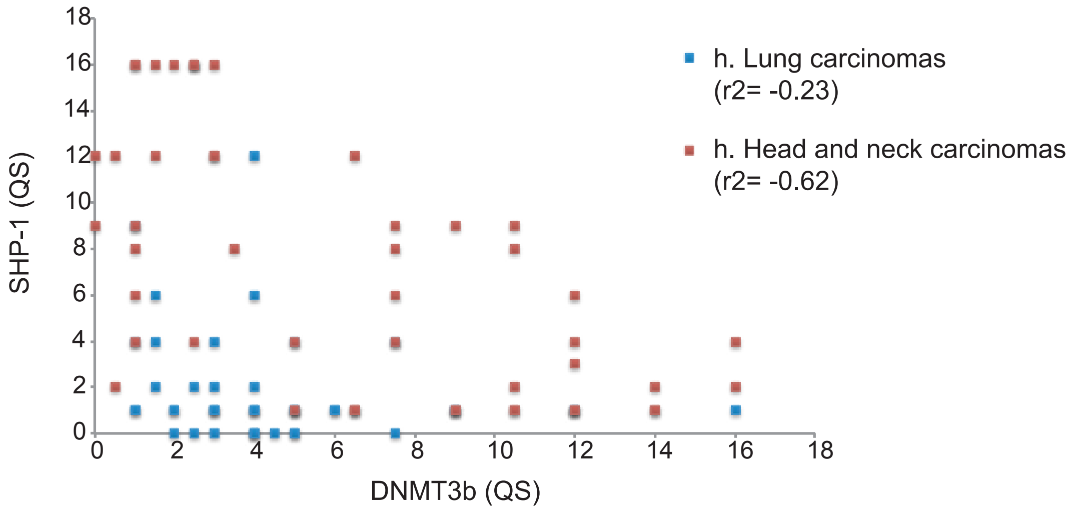

c

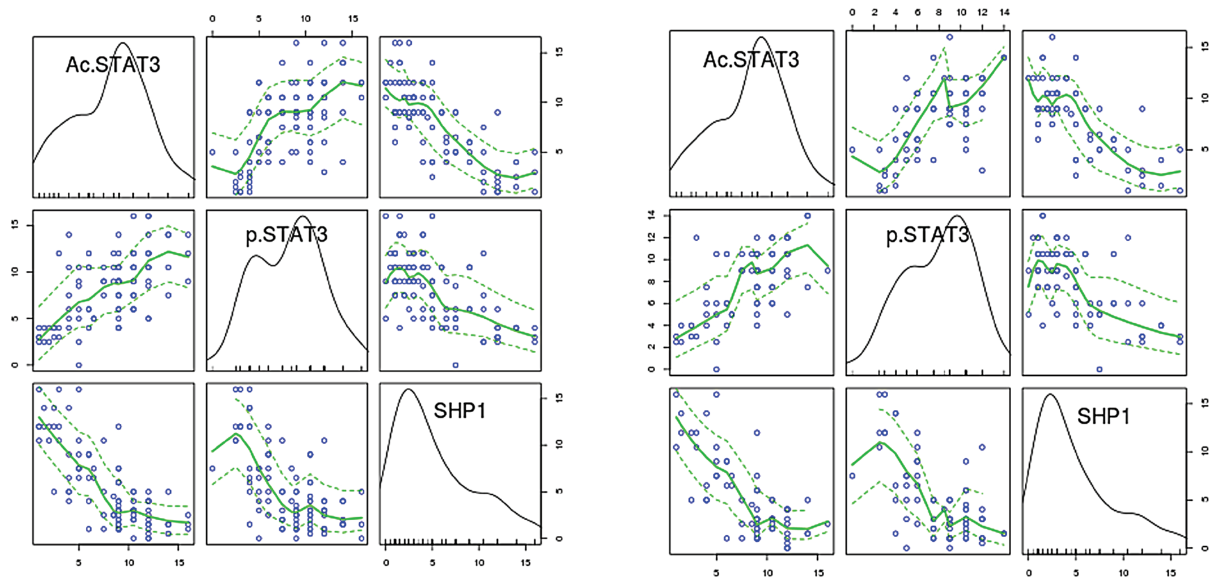

Supplementary Figure 6

Supplementary Figure 6

a) IgG control, DNMT3b and SHP-1 immunohistological staining in human head and neck (n=50) and lung (n=50) carcinomas. Scale bar 100µm. b) Plot of QS form DNMT3b (x axis) and SHP-1 (y axis) showing a negative correlation between DNMT3b and SHP-1 detection in lung (bleu) and head and neck (red) carcinomas. c) Scatter-plot matrix comparing experimental measurements and computed values from predictive models. Left figure shows all measurements as blue circles. Right plotted only values from the clean set of measurements. The regression models were represented as green curves, with dashed lines for uncertainties (based on P-value). Black curves were added to show the distribution of experimental measurements.

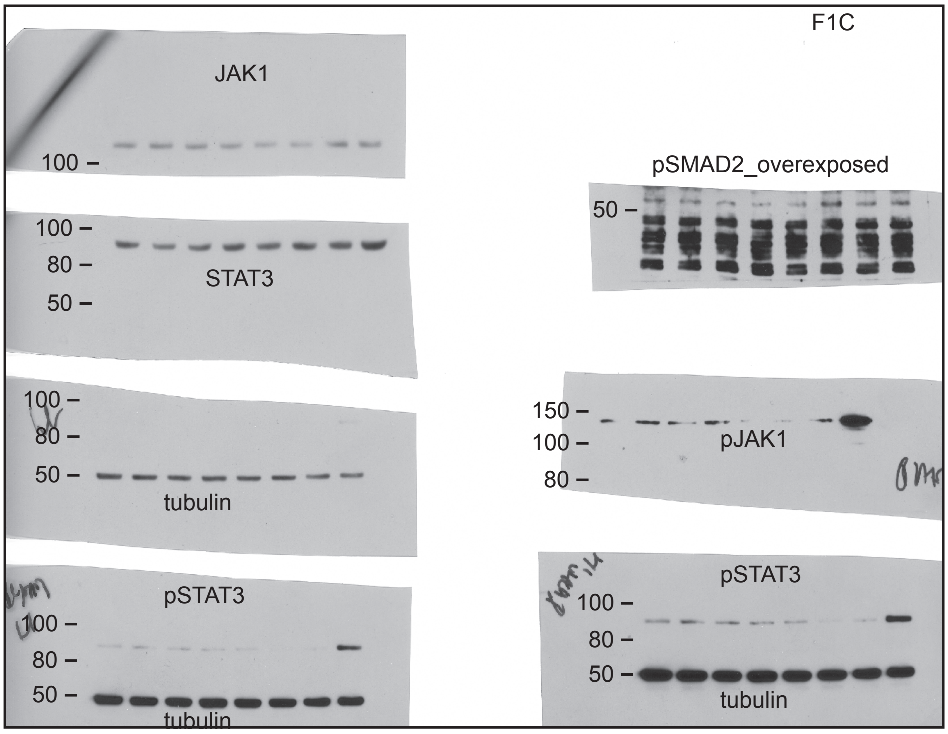

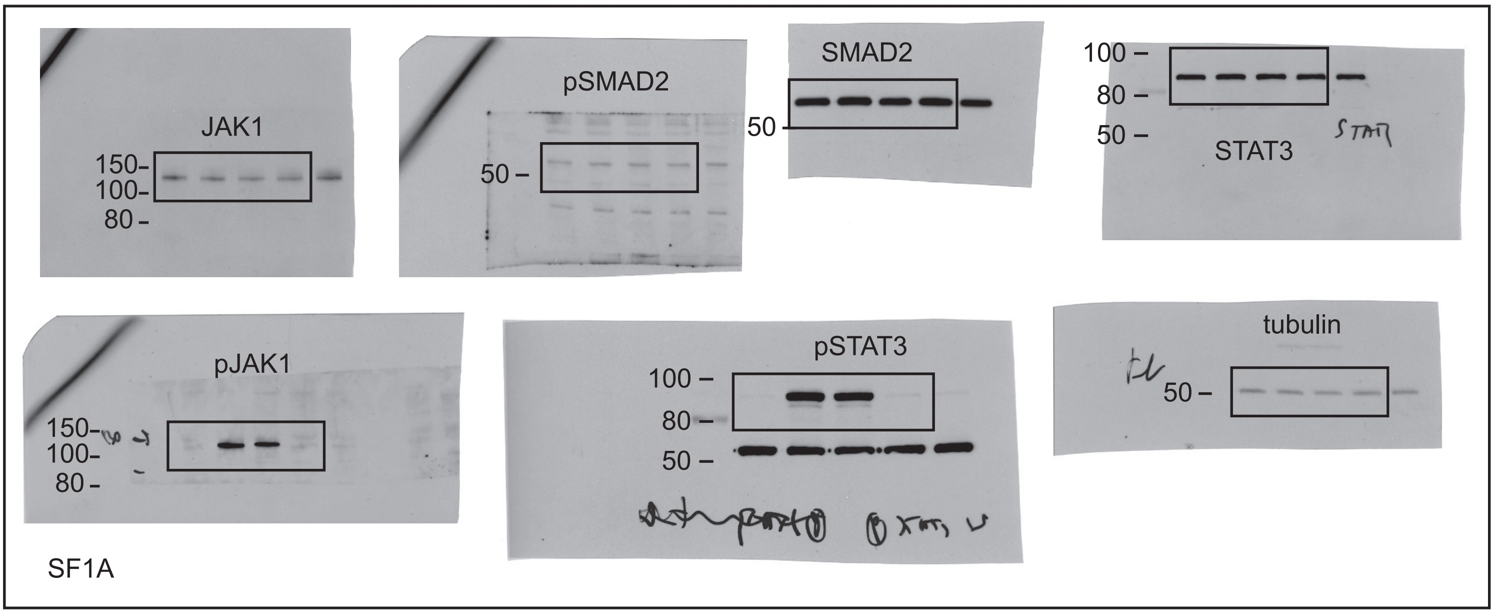

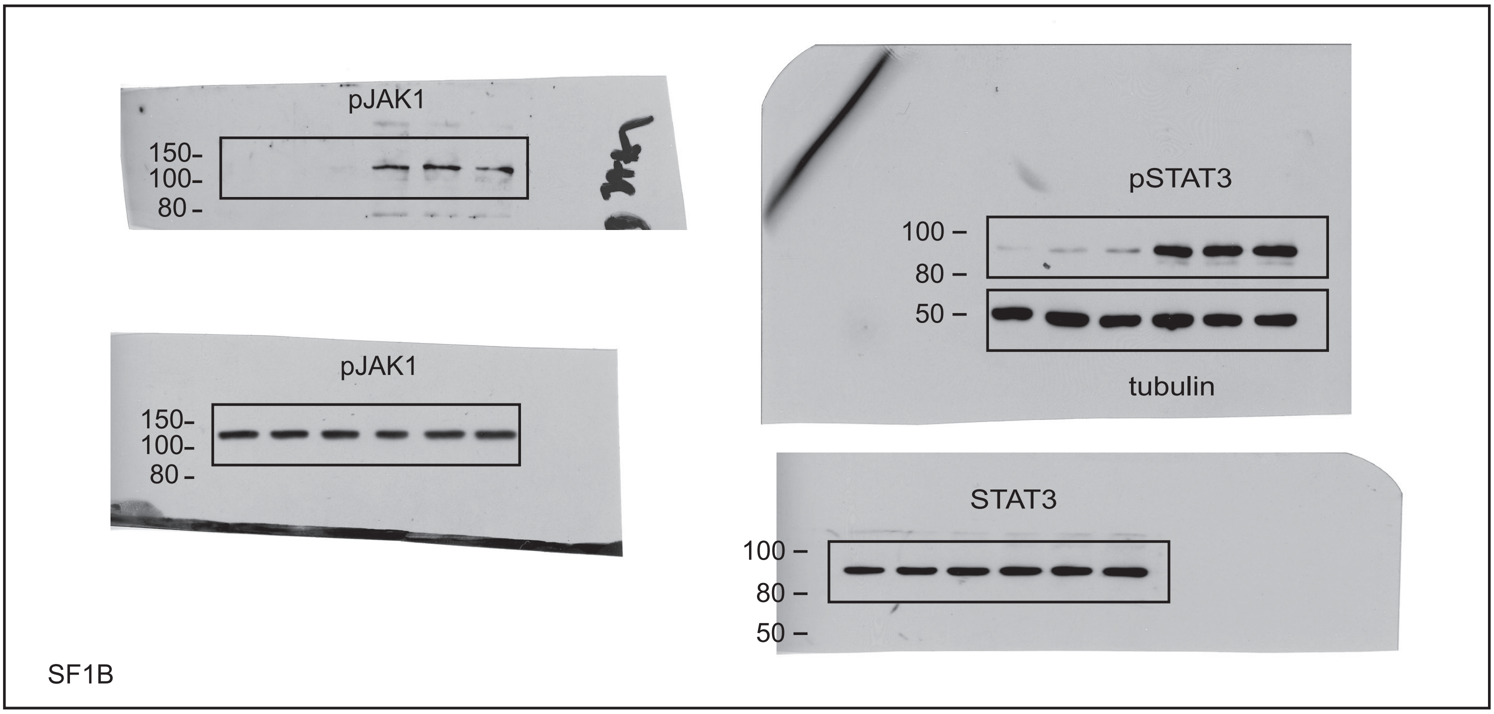

Supplementary Figure 7

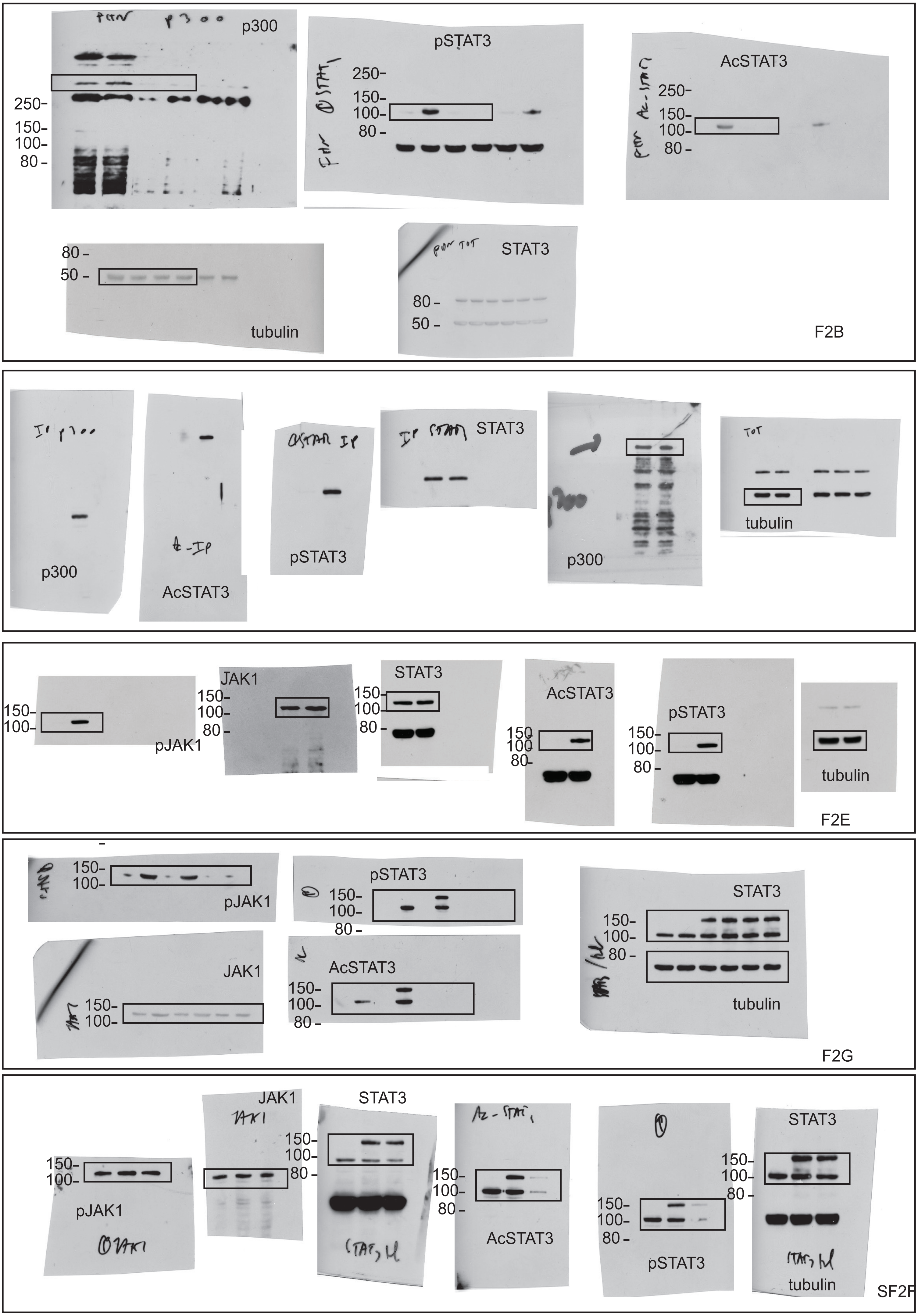

Supplementary Figure 7

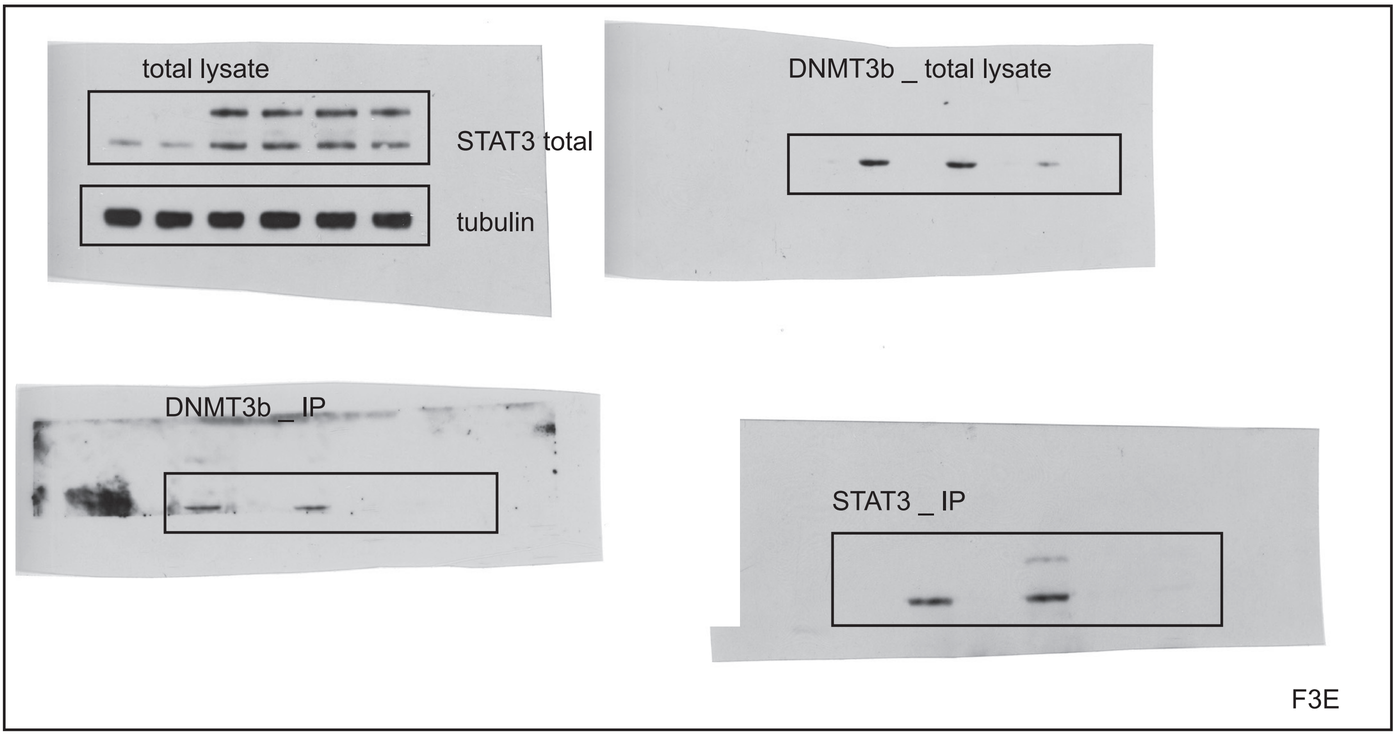

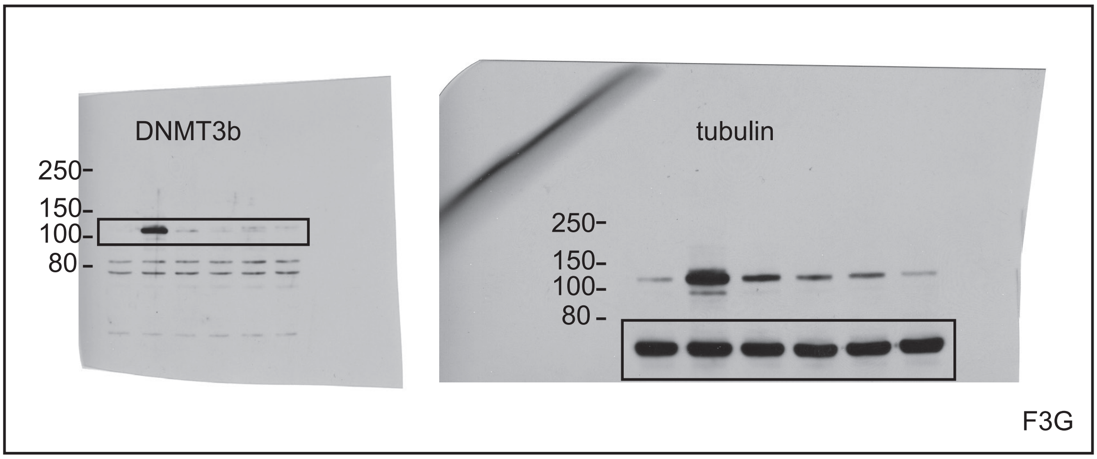

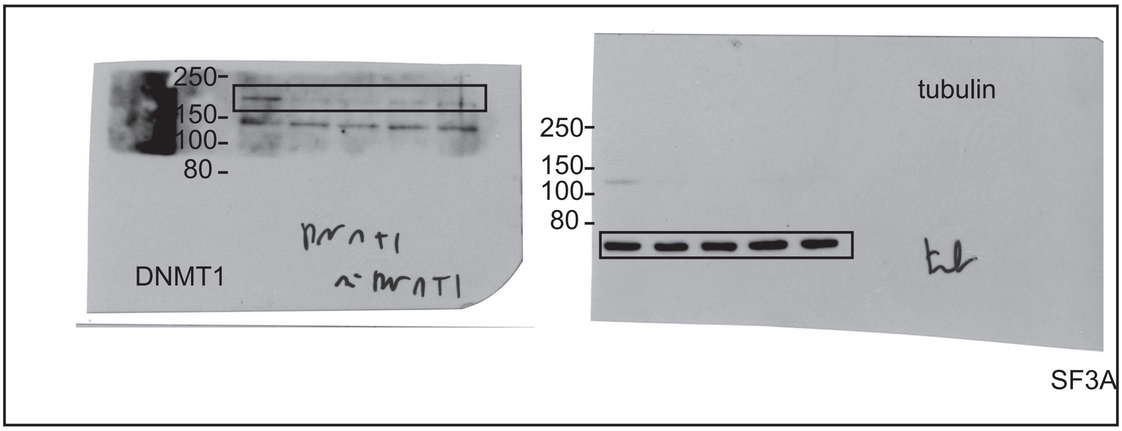

Supplementary Figure 7

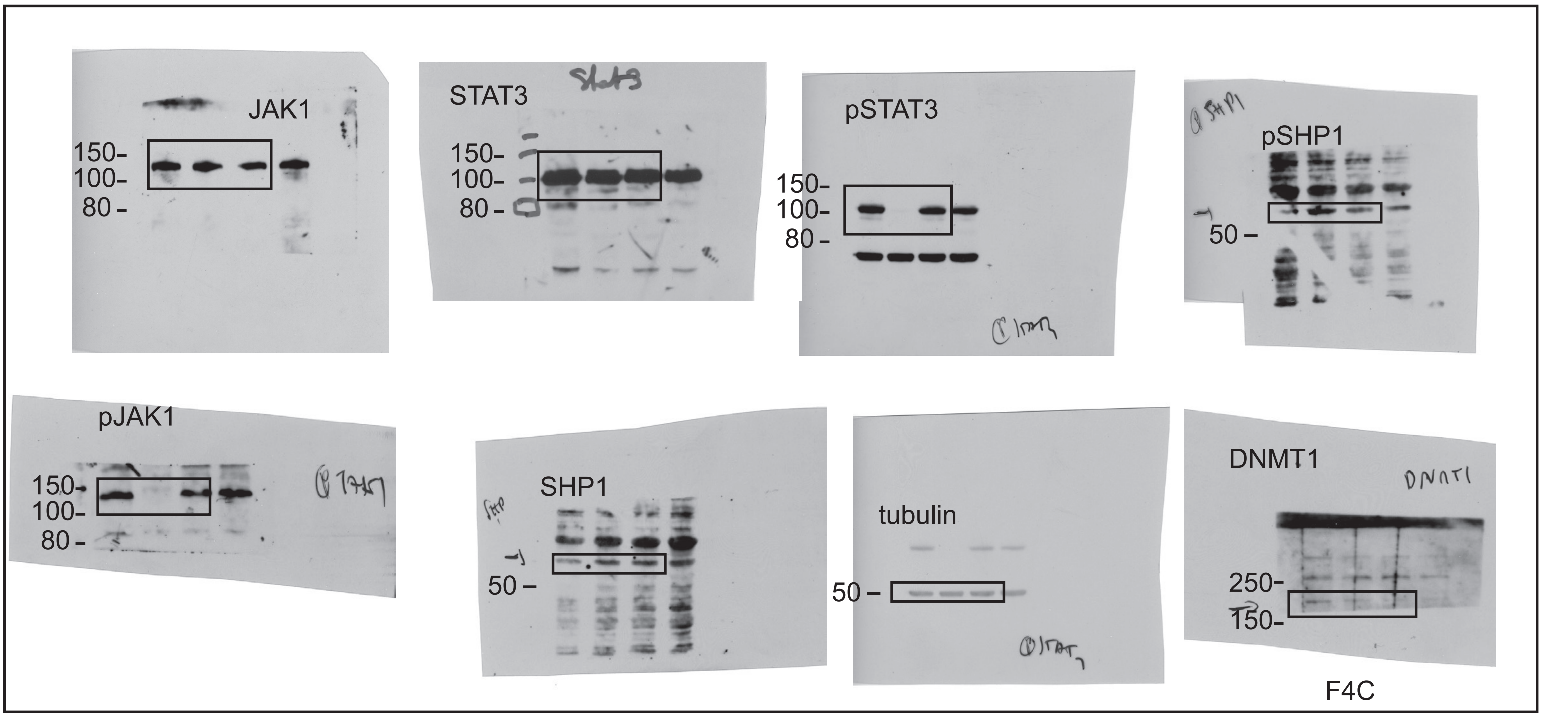

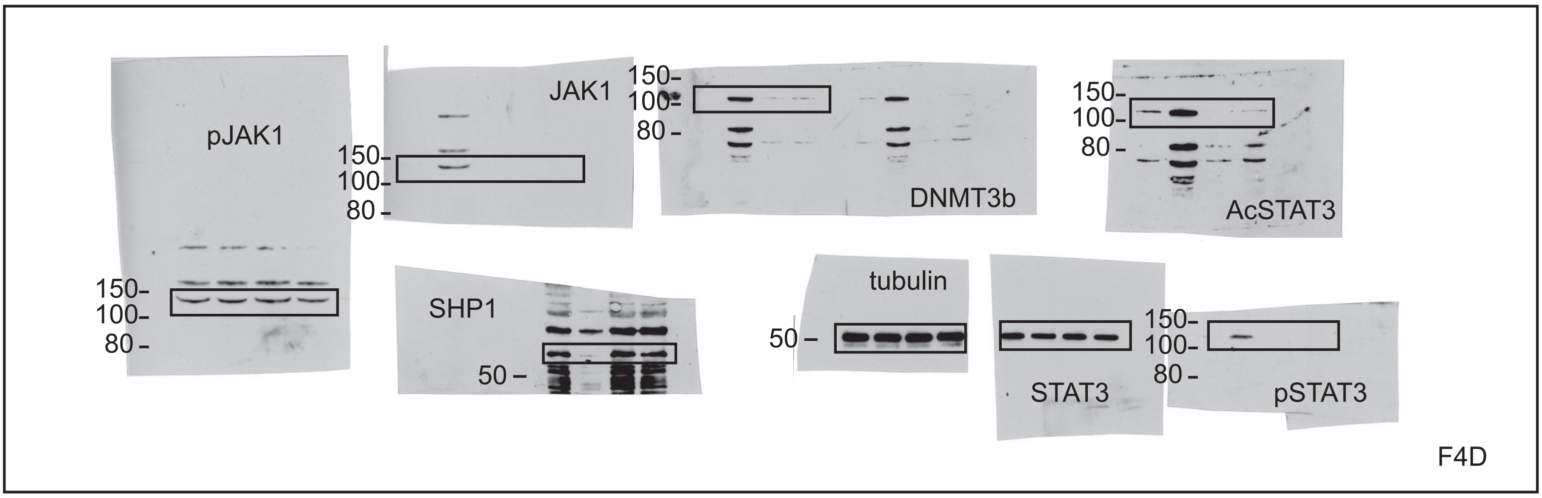

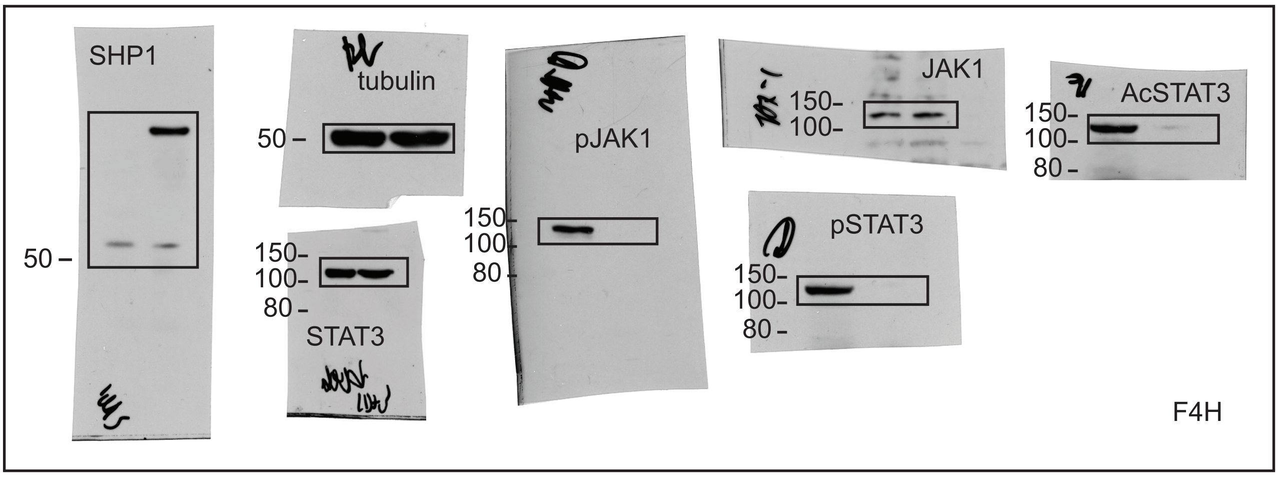

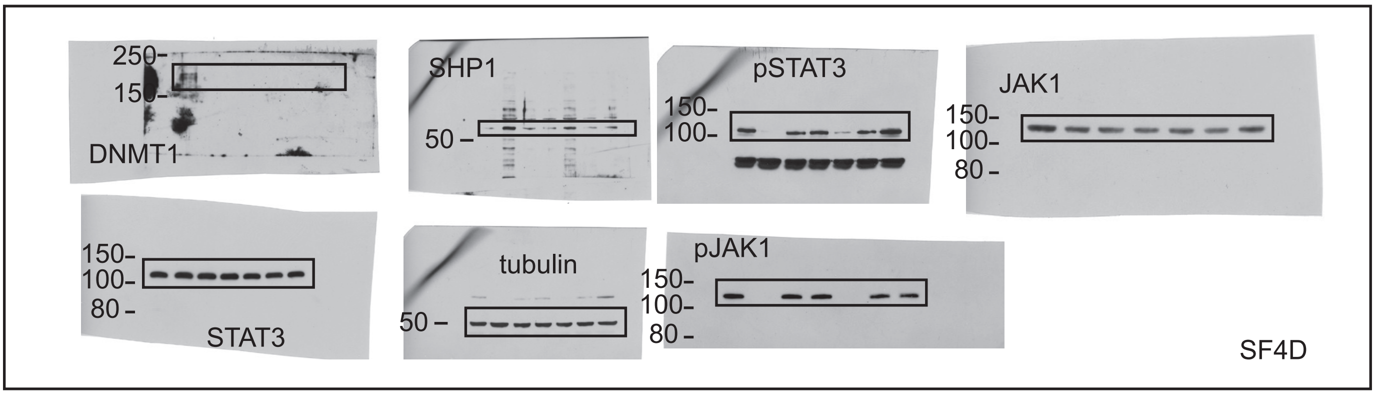

Supplementary Figure 7

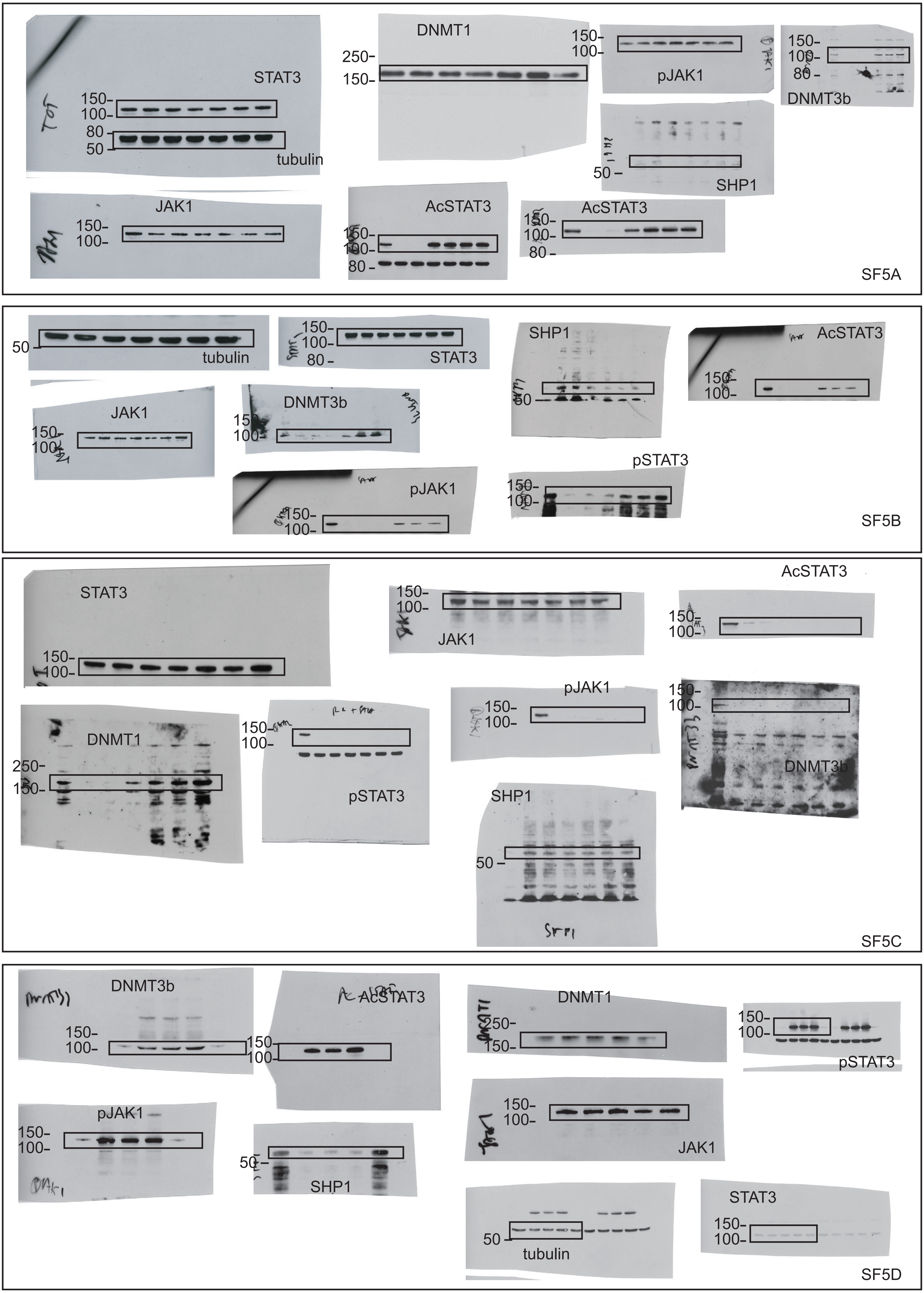

Supplementary Figure 7

Supplementary Figure 7

Original immunoblots for images used in the entire manuscript.

Classes	Names	Targets	Concentration (μM)	% of gel contraction			
				n=1	n=2	n=3	**mean**
**Veh.**	DMSO		15	53.19	55.31	48.93	**52.48**
**DNMT inhibitors**	5-Aza-2'-deoxycytidine		5	0	2.12	0	**0.709**
	Zebularine		15	4.25	2.12	4.25	**3.54**
	Decitabine		5	10.63	10.63	2.12	**7.80**
**HDAC inhibitors**	MC-1293	HDAC1	10	36.17	48.93	53.19	**46.09**
	Suberoyl bis-hydroxamic acid	HDAC1/3	15	44.68	53.19	57.44	**51.77**
	Scriptaid	HDAC1/3/8	0.5	63.82	53.19	55.31	**57.44**
	Nullscript	Negative control	0.5	57.44	51.06	48.93	**52.48**
	Valproic acid hydroxamate	Class 1/2	15	48.93	48.93	46.80	**48.22**
	Valproic acid	Class 1/2a	15	61.70	65.95	61.70	**63.12**
	CL-994	Class 1	10	36.17	46.80	53.19	**45.39**
	Saha	Class 1/2/4	0.5	59.57	61.70	59.57	**60.28**
	Phenylbutyrate-Na	Class 1/2/4	15	72.34	68.08	63.82	**68.08**
	Fluoro-Saha	Class 1/2/4	0.5	53.19	53.19	57.44	**54.60**
	Oxamflatin	Class 1/2	0.5	65.95	48.93	65.95	**60.28**
	NCH-51	Pan	5	44.68	48.93	53.19	**48.93**
	5-nitrosoquinolin-8-ol	Pan	5	48.93	57.44	53.19	**53.19**
	Trichostatin A	Class 1/2/4	0.1	61.70	57.44	59.57	**59.57**
	BML-210	Class 1/2	10	48.93	48.93	46.80	**48.22**
	Apicidin	Class 1/2	5	53.19	57.44	53.19	**54.60**
	M-344	Pan	0.5	44.68	42.55	44.68	**45.39**
	BML-281	Class 6	0.1	44.68	46.80	44.68	**45.39**
	ITSA-1	TSA inhibitor	15	57.44	53.19	51.06	**53.90**
**P300**	CTPB	Activator	15	78.72	74.46	74.46	**75.88**
	C646	Inhibitor	10	4.25	5.45	6.38	**5.36**
	Garcinol	Inhibitor	15	0	2.12	2.12	**1.41**
	Anacardic acid	Inhibitor	15	6.38	2.12	2.12	**3.54**
**Sirtuin**	Piceatannol	Activator	15	38.29	44.68	46.80	**43.26**
	Nicotinamide	Inhibitor	15	53.19	51.06	53.19	**52.48**
	Salermide	Inhibitor	15	38.29	36.17	46.80	**40.42**
	Sirtinol	Inhibitor	15	53.19	48.93	53.19	**51.77**
	Isonicotinamide	Sirt1 Activator	15	68.08	57.44	51.06	**58.86**
	Resveratrol	Sirt1 activator	15	31.91	36.17	27.65	**31.91**
	BML-278	Sirt1 activator	10	48.93	44.68	53.19	**48.93**
	Aminoresveratrol sulfate	Sirt1 activator	15	48.93	46.80	40.42	**45.39**
	Triacetylresveratrol	Sirt1 activator	15	53.19	55.31	51.36	**53.28**
	EX-527	Sirt1 inhibitor	10	34.04	25.53	23.40	**27.65**
	Splitomicin	Sirt2 inhibitor	5	53.19	48.93	51.06	**51.06**
	BML-266	Sirt2 inhibitor	5	51.06	51.06	48.93	**50.35**
	AGK2	Sirt2 inhibitor	10	51.06	53.19	51.06	**51.77**
	B2	Sirt2 inhibitor	15	59.57	53.19	48.93	**53.90**
**Others**	Suramine-6Na	Sirt1/5 inhibitor	15	53.19	48.93	53.19	**51.77**
	Butyrolactone 3	Histone acetyltrans-ferase Gcn5 inhibitor	15	57.44	53.19	48.93	**53.19**
	2,4-Pyridinedicarboxylic acid	Histone demethylase inhibitor	10	53.19	46.08	55.31	**51.77**
	BIX-01294	Histone methyltrans-ferase inhibitor	5	57.44	48.93	53.19	**53.19**
	Tranylcypromine hemisulfate	Lysine demethylase inhibitor	15	48.93	48.93	46.80	**48.22**

Supplementary Table 1

Supplementary Table 1

Epigenetic-dependent contractility screen; List, classes, concentration and percentage of gel contraction for each compounds used in this screen.

Supplementary Table 2: Correlation coefficients between variables

	Ac.STAT	*SHP1*	*p.STAT*
Ac.STAT	1.0000000	-0.6551330	0.7268858
SHP1	-0.6041013	1.0000000	-0.6185073
p.STAT	0.7579424	-0.6212431	1.0000000

Multivariate model : Ac.STAT ~ SHP1 + p.STAT

Variables	*Coefficients*	*P-value*
SHP1	-0.44081	< 0.0001
p.STAT	0.36421	0.00105
Intercept	7.57077	< 0.0001

Multivariate model : SHP1 ~ Ac.STAT+ p.STAT

Variables	*Coefficients*	*P-value*
Ac.STAT	-0.6661	< 0.0001
p.STAT	-0.3224	0.0206
Intercept	12.9979	< 0.0001

Multivariate model : p.STAT ~ Ac.STAT+ SHP1

Variables	*Coefficients*	*P-value*
Ac.STAT	0.3983	0.00105
SHP1	-0.2333	0.02064
Intercept	5.7869	< 0.0001

Supplementary Table 2

Values represent coefficients, in green using clean set (n=74), in black using whole set of measurements (n=100). Prediction models inferred from a multivariate logistic regression.
